# Human FUS is toxic via association with RNA polymerase II in *Drosophila*

**DOI:** 10.1038/s41419-026-08539-x

**Published:** 2026-03-14

**Authors:** Thomas G. Moens, Luca Biasetti, Wendy Scheveneels, Bradley N. Smith, Claire Troakes, Philip Van Damme, Caroline Vance, Ludo Van Den Bosch

**Affiliations:** 1 Department of Neurosciences, Experimental Neurology and Leuven Brain Institute (LBI), KU Leuven–University of Leuven, Leuven, Belgium; 2 VIB, Center for Brain & Disease Research, Laboratory of Neurobiology, Leuven, Belgium; 3 Maurice Wohl Clinical Neuroscience Institute and the Institute of Psychiatry, Psychology and Neuroscience, King’s College London, London, UK; 4 Department of Neurology, University Hospitals Leuven, Leuven, Belgium; 5 Present Address: CRUK Scotland Institute, Garscube Estate, Glasgow, UK

**Keywords:** Amyotrophic lateral sclerosis, Cellular neuroscience

## Abstract

The RNA-binding protein FUS is commonly mutated in familial cases of amyotrophic lateral sclerosis (ALS-FUS), where it forms cytoplasmic inclusions. In addition, non-mutated FUS is a constituent component of protein inclusions in approximately 5–10% of cases of frontotemporal lobar degeneration (FTLD). Overexpression of wild-type human FUS is toxic to *Drosophila* neurons, preventing normal development and shortening lifespan in adults. In this study, we demonstrated that removal of the nuclear localisation sequence (NLS) of FUS, a common consequence of ALS-associated mutations, unexpectedly prevents toxicity in *Drosophila* models despite inducing FUS cytoplasmic mislocalisation. Using novel flies capable of expressing mGFP-tagged FUS, we found that FUS forms dynamic protein granules in *Drosophila* nuclei and does not form insoluble aggregates. FUS and other FET-family paralogues interact with the repetitive disordered C-terminal domain (CTD) of the large subunit of RNA polymerase II (Polr2A). Using flies that have variable CTD repeat lengths, we demonstrated that FUS genetically interacts with the Polr2A CTD to induce toxicity. Finally, we demonstrated that this association with Polr2A could be relevant to human disease, finding that inclusion-bearing neurons of individuals with FUS-positive FTLD, but not ALS-FUS, show cytoplasmic mislocalisation of POLR2A (the Polr2A human orthologue). Together, these results imply that FUS can have a nuclear mechanism of toxicity when overexpressed in animal models. This toxicity occurs via interaction with RNA polymerase II and aberrant interaction between FUS and POLR2A may be involved in the pathogenesis of FTLD.

## Introduction

Amyotrophic lateral sclerosis (ALS) is an adult-onset neurodegenerative disease that results in loss of both upper and lower motor neurons, leading to progressive muscle weakness [1, 2]. Death usually occurs within 3 years of diagnosis due to respiratory failure [1, 2]. Up to 50% of ALS patients develop behavioural or cognitive dysfunction, while around 15% of ALS patients meet the diagnostic criteria for frontotemporal dementia (FTD), linking the two diseases as part of a spectrum [3, 4]. FTD is characterised by behavioural alterations or primary progressive aphasia [5]. FTD symptoms are caused by neurodegeneration of the frontal and temporal cortices and when confirmed pathologically, the disease is referred to as frontotemporal lobar degeneration (FTLD). Both ALS and FTLD currently lack effective treatment options.

In 2009, it was discovered that mutations in the DNA/RNA binding protein Fused In Sarcoma (FUS) are a cause of familial ALS [6, 7]. Mutations in FUS account for approximately 1–5% of familial ALS cases and <1% of sporadic ALS cases [8].

In ALS patients with FUS mutations, FUS becomes mislocalised from the nucleus to the cytoplasm, where it forms inclusions in neurons and glial cells [7, 9, 10]. The most commonly observed mutations are in the C-terminal proline-tyrosine nuclear localisation sequence (NLS) [10–13]. Mutations in the NLS result in a failure of nuclear import [10–13]. The severity of mutation-induced FUS mislocalisation correlates with an earlier age of onset in patients [11]. FUS mislocalisation is thought to lead to a cytoplasmic gain-of-function rather than a nuclear loss-of-function, as knockout of FUS in mice does not produce ALS-like phenotypes [14, 15].

FUS, along with its two paralogues, TAF15 and EWS, constitute the FET-family of DNA/RNA binding proteins [16]. Inclusions of FET-family protein members are observed in the brains of around 5–10% of patients with frontotemporal lobar degeneration (FTLD-FET) [10, 17]. Recent evidence suggests that these inclusions may predominantly be formed of an amyloid core of TAF15, with FUS and other proteins such as nuclear import receptors recruited later [18]. FTLD-FET pathology normally occurs in the absence of FUS mutations and inclusions can be both cytoplasmic and intranuclear [19, 20]. Given these differences in the routes of FUS aggregation, the localisation of FUS inclusions within the tissue and their composition, it is unclear whether dysfunctional FUS or other FET family members are toxic via the same mechanism in ALS and FTLD [10].

We and others have previously shown that overexpression of wild-type or ALS-mutant human FUS is toxic to *Drosophila* neurons. Overexpressed FUS inhibits normal development, or when expressed in adults, results in reduced lifespan and motility [21–25]. Recently, we mapped the domains required for human FUS toxicity in *Drosophila*, finding that deletion of the prion-like (QGSY) domain of the protein or the arginine-rich regions of the protein abrogates toxicity [25]. In the course of these experiments, we observed that deletion of the NLS of the FUS protein unexpectedly appears to reduce toxicity.

Here, we explored this phenomenon further: we demonstrated that cytoplasmic mislocalisation of overexpressed FUS protein is protective in *Drosophila* models. We found that overexpressed wild-type human FUS protein localised to dynamic intranuclear granules, which colocalise with the large subunit of RNA polymerase II (Polr2A). Reducing the length of the repetitive C-terminal domain (CTD) for Polr2A lessened FUS toxicity. This suggests that FUS is toxic via association with this repetitive region. Finally, we observed that the human orthologue of Polr2A (POLR2A) undergoes cytoplasmic mislocalisation in post-mortem tissue from patients with FTLD-FET, suggesting that this interaction is relevant to the human disease state.

## Results

### Human FUS is toxic via a nuclear mechanism in *Drosophila*

**Fig. 1:**
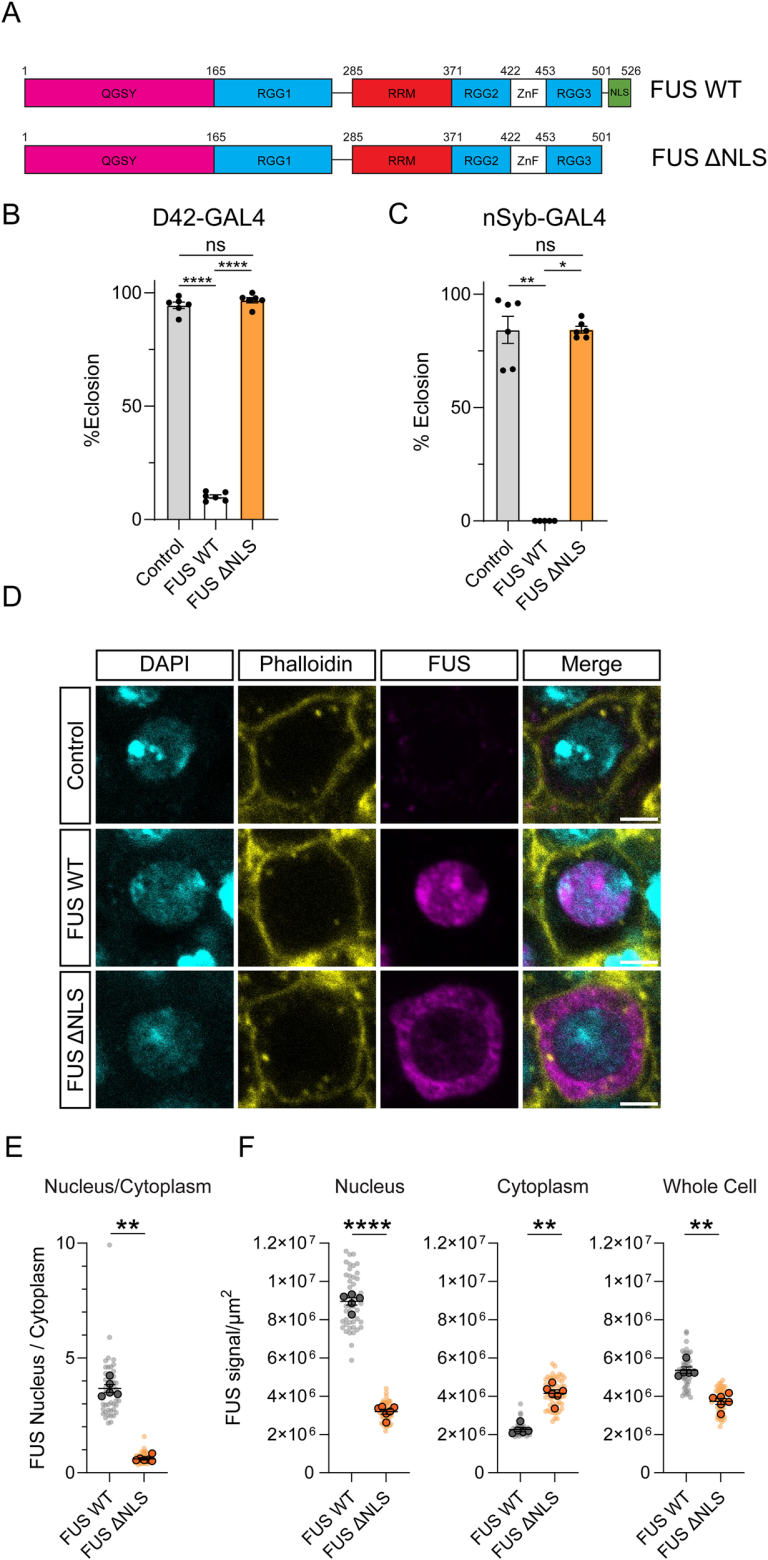
Toxicity of overexpressed human FUS is mitigated by deletion of the NLS in *Drosophila.* **A** Domain diagram showing wild-type human FUS and the FUS ΔNLS, which is truncated after amino acid 501. These proteins were overexpressed in *Drosophila* neurons in the following experiments. **B** FUS fly lines were crossed to the motor neuron driver D42-GAL4 at 25 °C and eclosion was assessed. Driver alone (w-;; D42-GAL4/+) was used as a control. ****P < 0.0001, ns=*P* > 0.05, Tukey’s multiple comparisons test after one-way ANOVA (F (2, 15) = 1819, *P* < 0.0001). *n* = 6 vials per condition, bars are mean ± SEM, individual data points are shown. **C** FUS fly lines were crossed to nSyb-GAL4 at 25 °C and eclosion was assessed. Driver alone (w-;;nSyb-GAL4/+) was used as a control. *P = 0.0242, **P = 0.0086, ns=*P* > 0.05, Dunn’s multiple comparisons test after Kruskal-Wallis test (H(2) = 10.37, *P* = 0.0015). *n*(Control), *n*(ΔNLS) = 6 vials, *n*(WT) = 5 vials. Bars are mean ± SEM, individual data points are shown. **D** Confocal microscopy images of larval motor neurons expressing FUS constructs. DAPI and phalloidin were used to mark the nucleus and the edge of the cell body, respectively. FUS was detected using an N-terminal region-targeting monoclonal antibody. A driver-only control was used to confirm antibody specificity. WT FUS protein was predominantly nuclear, while ΔNLS is mislocalised to the cytoplasm. Scale bar = 3 µm. **E** Quantification of the ratio of nuclear/cytoplasmic FUS protein in FUS WT and FUS ΔNLS expressing larvae. Plots show values from individual cells (small data points) as well as the average from each individual central nervous system (CNS) (large data points). Number of individual neurons analysed: (WT) = 47, *n*(ΔNLS) = 55. Number of individual CNSs analysed: *n*(WT) = 5, *n*(ΔNLS) = 6. **P = 0.0043, two-tailed Mann-Whitney test comparing CNS averages. Bars are mean ± SEM of CNS averages. **F** Plots showing the FUS signal intensity normalised to area (µm^2^) for the nucleus, cytoplasm and whole cell. Plots show values from individual cells (small data points) as well as the average from each CNS (large data points). Number of individual neurons analysed: (WT) = 47, *n*(ΔNLS) = 55. Number of individual CNSs analysed: *n*(WT) = 5, *n*(ΔNLS) = 6. ****P < 0.0001, two-tailed t-test comparing CNS averages. **P = 0.0043, two-tailed Mann-Whitney test comparing CNS averages. Bars are mean ± SEM of CNS averages.

Numerous studies have reported that overexpression of wild-type human FUS is toxic to *Drosophila* neurons [21, 22, 24, 25]. Previously, we generated a range of FUS deletions and expressed them in *Drosophila* motor neurons as part of a screen to identify domains of the protein important for neurotoxicity [25]. Consistent with previous results, we found that motor-neuron-specific overexpression of wild-type human FUS protein (FUS WT) resulted in pupal lethality prior to eclosion (Fig. 1A, B). We observed that the FUS-induced eclosion defect was prevented when FUS was truncated after amino acid 501, leading to loss of the NLS of the protein (FUS ΔNLS) (Fig. 1A, B). We observed similar results when FUS was pan-neuronally overexpressed using the nSyb-GAL4 driver line (Fig. 1C). We expected that removal of the NLS would cause FUS to become cytoplasmically mislocalised. To assess this, we expressed WT and ΔNLS FUS in larval motor neurons and immunostained for FUS using an antibody raised against the N-terminal region of the protein (Fig. 1D). We used fluorescently labelled phalloidin to visualise the edges of the cell soma. As expected, while WT FUS was predominantly intranuclear, ΔNLS FUS was mislocalised to the cytoplasm (Fig. 1D). When quantified, this resulted in a significant reduction in the nuclear/cytoplasmic ratio of FUS in ΔNLS expressing motor neurons compared to WT FUS (Fig. 1E). We assessed relative FUS signal in each compartment (Fig. 1F). In FUS ΔNLS flies, we observed a significant reduction in nuclear FUS level, as well as an overall lower level of FUS protein at the level of the whole cell (Fig. 1F). Nevertheless, we observed a significantly greater signal in the cytoplasm (Fig. 1F). This demonstrates that, as in mammals, the NLS is required for FUS nuclear import in *Drosophila*. Given that the majority of WT FUS localises to the nucleus and that there is a substantially higher amount of cytoplasmic FUS in the ΔNLS condition compared to WT, we conclude that FUS is toxic to *Drosophila* neurons via a predominantly nuclear mechanism.

**Fig. 2:**
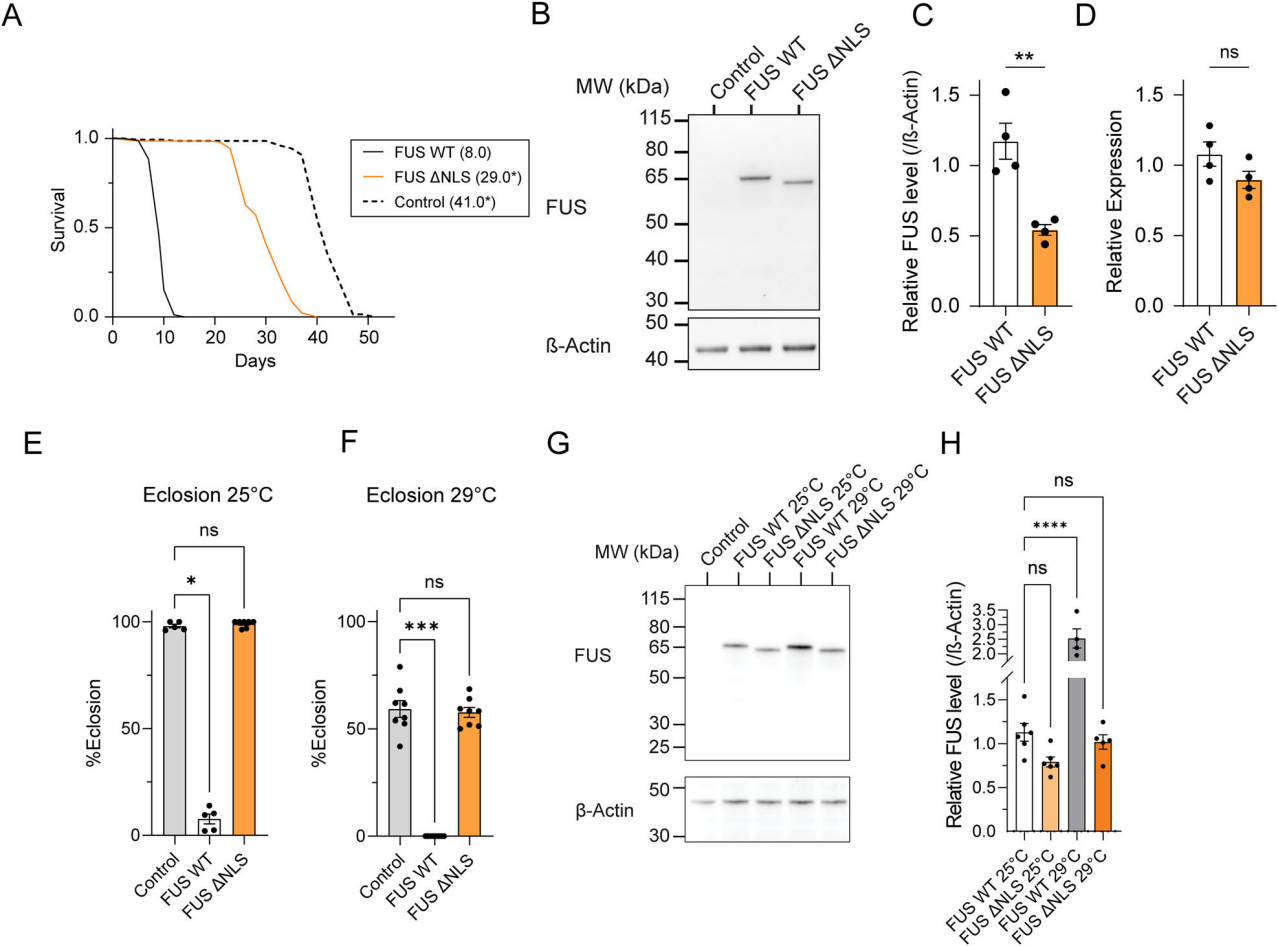
Expressing ΔNLS FUS at a level equivalent to wild-type does not induce toxicity. **A** Lifespans of males expressing FUS pan-neuronally, with expression induced after development, at 29 °C. Control flies lack a UAS-FUS transgene (w-; tubP-GAL80^ts^ /+; nsyb-GAL4/+). Median lifespans are given in brackets. *P < 0.05 compared to FUS WT (Log-rank test) *P*(ΔNLS) = 3.05E-58, *P*(control)=1.69E-60. *n*(Control)=144, *n*(WT) = 89, *n*(ΔNLS) = 142 males per group. Full details and statistical tests are given in Supplementary Tables 1 and 2. **B** Representative western blot of head lysates from flies expressing FUS WT or FUS ΔNLS pan-neuronally with expression induced after development at 29 °C. Lysates from control flies, lacking a FUS transgene, were used to confirm antibody specificity. **C** Quantification of protein levels of FUS normalised to β-Actin using western blotting. **P = 0.0034, two-tailed t-test. *n* = 4 biological replicates per condition, bars are mean ± SEM, individual data points are shown. **D** Transcript abundance of FUS WT and FUS ΔNLS was assessed using qPCR with primers designed against the N-terminal coding region of FUS. ns=*P* > 0.05 (*P* = 0.1362), two-tailed t-test. *n* = 4 biological replicates per condition, bars are mean ± SEM, individual data points are shown. **E** FUS fly lines were crossed to the motor neuron driver D42-GAL4 at 25 °C eclosion assessed. Driver alone (w-;;D42-GAL4/+) was used as control. *P = 0.0327, ns=*P* > 0.05, Dunn’s multiple comparisons test, after Kruskal-Wallis test (H(2) = 11.17, *P* = 0.0006). *n*(Control)=5, *n*(FUS WT) = 5, *n*(FUS ΔNLS) = 7, vials. **F** FUS fly lines were crossed to the motor neuron driver D42-GAL4 at 29 °C eclosion was assessed. Driver alone (w-;;D42-GAL4/+) was used as control. ***P = 0.0006, ns=*P* > 0.05, Dunn’s multiple comparisons test, after Kruskal-Wallis test (H(2) = 16.03, *P* = 0.0003). *n*(Control)=8, *n*(FUS WT) = 8, *n*(FUS ΔNLS) = 8, vials. bars are mean ± SEM, individual data points are shown (**G**) Representative western blot of central nervous system lysates from L3 *Drosophila* larvae expressing FUS WT or FUS ΔNLS in motor neurons using the D42-GAL4 driver. Lysates from control larvae, lacking a FUS transgene (w-;;D42-GAL4/+) were used to confirm antibody specificity. **H** Quantification of FUS protein levels in the central nervous system lysates from L3 *Drosophila* larvae. ****P < 0.0001, ns=*P* > 0.05, Dunnett’s multiple comparisons test compared to FUS WT 25 °C following one-way ANOVA (F(3,17) = 25.06, *P* < 0.0001). *n*(FUS WT 25 °C) = 6, *n*(FUS ΔNLS 25 °C) = 6, *n*(FUS WT 29 °C) = 4, *n*(FUS ΔNLS 29 °C) = 5, independent dissections. bars are mean ± SEM, individual data points are shown.

We next expressed FUS in adult neurons using the pan-neuronal driver nSyb-GAL4. To avoid expression during development, we included a ubiquitously expressed temperature-sensitive-GAL80 (tubP-GAL80^TS^) transgene, which acts as a temperature-sensitive repressor of the GAL4/UAS system. We reared the flies at 18 °C, a temperature that represses GAL4-mediated transcription, before shifting them to 29 °C as adults to induce expression. While WT FUS-expressing flies died with a median lifespan of 8.0 days, ΔNLS flies lived for 29.0 days, a significant extension (Fig. 2A). ΔNLS flies were still short lived compared to controls, possibly implying a mild cytoplasmic toxicity (Fig. 2A).

We performed immunoblotting to assess the level of WT and ΔNLS FUS using an antibody directed to the N-terminal region of these proteins (Fig. 2B). We observed a significantly lower FUS protein abundance in ΔNLS flies (Fig. 2B, C). qPCR of the FUS transcript confirmed that these differences in protein abundance were not due to differences in transcript levels (Fig. 2D), implying that the differences in FUS protein levels occur translationally or post-translationally.

To assess whether ΔNLS FUS mislocalises in the adult fly brain, we expressed WT and ΔNLS FUS in the insulin-producing neurons of the adult *Drosophila* brain. We chose these cells as they have large, well-demarcated nuclei and cell bodies, which we visualised via the use of a membrane-localised eGFP transgene. We, again, included a tubP-GAL80^ts^ transgene to prevent expression during development. Like in larvae, WT FUS was predominantly intranuclear and ΔNLS FUS was more abundant in the cytoplasm (Supplementary Fig. 1A). This finding was confirmed by quantifying the ratio of nuclear to cytoplasmic FUS, which was significantly reduced in the ΔNLS flies (Supplementary Fig. 1B). We again observed a significantly higher level of cytoplasmic FUS in the ΔNLS flies, with a trend towards a reduction of FUS level in the nucleus (Supplementary Fig. 1C). In these insulin-producing neurons, the level of FUS protein across conditions was equivalent at the level of the whole cell (Supplementary Fig. 1C).

Given that we have tended to observe a lower level of ΔNLS FUS protein compared to WT, we aimed to express these proteins at more equivalent levels to rule out an effect of FUS level on toxicity. The *Drosophila* GAL4/UAS gene expression system is temperature sensitive, with higher temperatures inducing higher expression [26]. When expressed in larval motor neurons at either 25 °C or 29 °C the eclosion of ΔNLS FUS expressing flies is not significantly different from controls, although we generally observed a reduced eclosion rate at 29 °C potentially due to stress or toxicity of the driver (Fig. 2E). Comparing the abundance of FUS protein across temperatures, we saw that ΔNLS FUS was somewhat lower than WT FUS at 25 °C (although this didn’t reach statistical significance) (Fig. 2F, G). However, the level of ΔNLS FUS at 29 °C was more equivalent to the level of WT FUS at 25 °C (Fig. 2G). This suggests that even when ΔNLS FUS and WT FUS are present in motor neurons at similar levels, ΔNLS FUS does not cause toxicity, while WT FUS does. This is in line with previous studies, which have expressed WT and ΔNLS FUS to the same protein level using a random transgene insertion approach and also observed ΔNLS FUS to be less toxic than WT [24].

Together, our results show that, in both larval and adult neurons, ΔNLS FUS is more abundant in the cytoplasm than WT. Even when the generally lower level of ΔNLS FUS is corrected for, toxicity does not occur. Our results in larval and adult flies, therefore, support a nuclear mechanism of FUS toxicity.

### FUS forms dynamic structures in the *Drosophila* nucleus

**Fig. 3:**
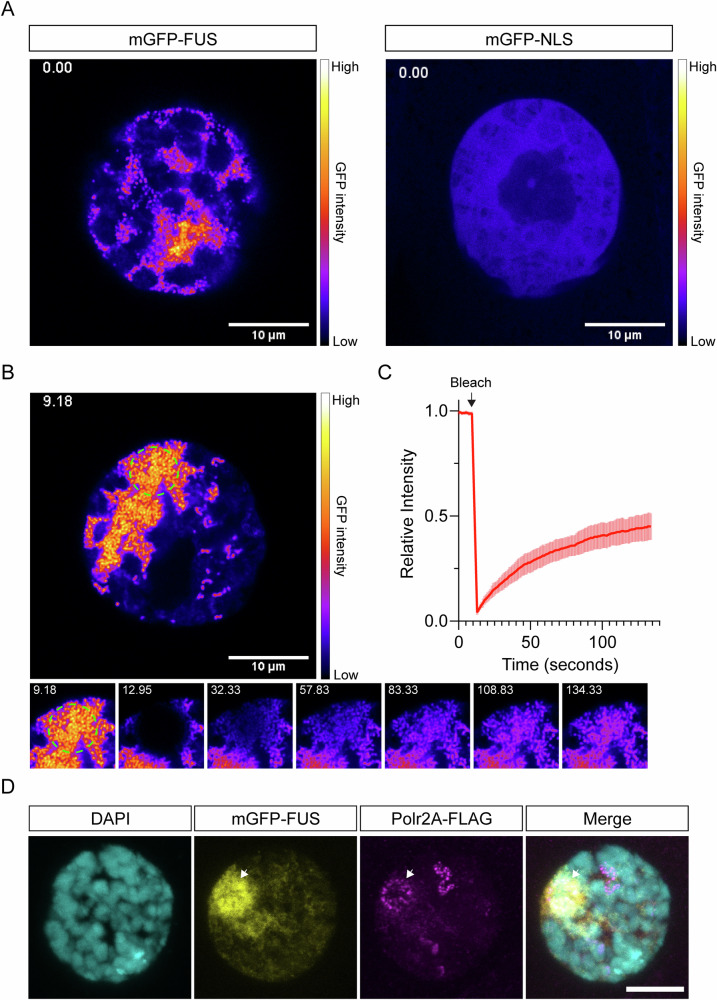
mGFP-tagged FUS forms dynamic granules within the nucleus. **A** Stills from Supplementary movie 1 (mGFP-FUS) and movie 2 (mGFP-NLS). Scale for the FIRE look-up table is shown. Scale bar = 10 µm. **B** Still from Supplementary movie 3, showing the time point immediately prior to photobleaching (9.18 s). The region to undergo bleaching is indicated by the green circle. Below are cropped stills from supplementary movie 3, showing the bleached region immediately before and after recovery from photobleaching. Time (in seconds) is shown for each still. **C** The recovery after photobleaching was assessed for multiple nuclei (*n* = 11 nuclei from 3 animals). The mean signal intensity normalised to the maximum recorded intensity is shown. Line is mean ± SD. The calculated recovery half-time is 30.35 s ± 7.97 s (SD). **D** A fly stock carrying an endogenously C-terminally flag-tagged Polr2A subunit (Polr2A-FLAG) was combined with mGFP-FUS expression. Salivary glands immunostained for FLAG show partial colocalisation with regions of dense mGFP-FUS abundance (arrow).

To assess the behaviour of FUS in the nucleus, we generated transgenic flies that can inducibly express WT FUS tagged with N-terminal monomeric GFP (mGFP). As a control, we generated a line that can express mGFP fused to the human FUS NLS (mGFP-NLS). To examine the behaviour of FUS in the *Drosophila* nucleus, we expressed mGFP-FUS in larval salivary gland cells using the fkh-GAL4 driver. We chose this tissue as the nuclei are very large (~20 µm in diameter) and have large, compacted chromosomes that allowed us to assess association with DNA. Using live confocal imaging of ex vivo tissue, we observed that mGFP-FUS forms punctate granular structures in the space around the chromosomes (Fig. 3A and Supplementary Movie 1). We did not observe the mGFP-NLS control protein form puncta; it remained diffuse in the nucleus (Fig. 3A and Supplementary Movie 2). We noted that mGFP-FUS was generally more abundant in the nucleus than the mGFP-NLS control (Supplementary Fig. 2A). However, the GFP signal intensity in individual nuclei expressing mGFP-FUS or mGFP-NLS partially overlapped and while we observed mGFP-FUS puncta in every nucleus, we failed to observe mGFP-NLS puncta in any nuclei, suggesting that this effect is not an artefact of higher mGFP-FUS abundance.

We confirmed that mGFP-tagged WT FUS was toxic, while mGFP-NLS allowed for normal eclosion when expressed in *Drosophila* motor neurons (Supplementary Fig. 2B). Compared to untagged FUS, mGFP-tagged FUS appears to be slightly less toxic (Fig. 1B vs. Supplementary Fig. 2B). To ensure that the mGFP tag wasn’t changing the localisation and/or behaviour of FUS, we overexpressed untagged wild-type FUS in salivary gland nuclei. Using immunostaining, we observed that untagged wild-type FUS protein formed puncta in the nucleus, suggesting that both mGFP-tagged and untagged wild-type FUS proteins can form granules in the nucleus (Supplementary Fig. 2C).

We next wondered whether FUS forms nuclear granules in neurons. When expressed in adult *Drosophila* neurons mGFP-FUS forms puncta within the nucleus while mGFP-NLS remains diffuse (Supplementary Fig. 2D), consistent with our salivary gland data. To assess whether FUS puncta were positive for mRNA, we performed fluorescent in situ hybridisation with an oligo-dT probe, which targets polyadenylated mRNA. We did not observe FUS-positive puncta in salivary glands to be strongly positive for mRNA (Supplementary Fig. 2E), although it is not possible to rule out an association with non-polyadenylated pre-mRNA.

We frequently observed that large patches of mGFP-FUS WT granules formed at one side of the nucleus. As these patches were relatively stationary, we performed fluorescent recovery after photobleaching on approximately half of the patch area. Upon photobleaching, we observed a recovery in fluorescent signal over a time scale of seconds, suggesting that there is a dynamic exchange of mGFP-FUS molecules between the FUS granules (Fig. 3B, C and Supplementary Movie 3).

The dynamic nature of the observed FUS granules is unexpected, given that it has previously been reported that WT FUS protein is predominantly insoluble in RIPA buffer when expressed in *Drosophila* neurons, implying that it forms protein aggregates [22]. Surprisingly, we found that this RIPA insolubility is temperature dependent. When WT FUS was extracted from the brains of flies in a room temperature buffer, it was soluble. However, FUS became insoluble when the tissue lysate was prepared using cold RIPA buffer on ice (Supplementary Fig. 2F). Therefore, previous studies may have observed FUS to be insoluble due to this unusual temperature-sensitive effect.

A number of studies have suggested that FUS is able to interact with the mRNA transcriptional apparatus via its association with RNA polymerase II [27–30]. We assessed whether mGFP-tagged FUS colocalised with the large subunit of RNA polymerase II (Polr2A) by crossing FUS-expressing flies to a line where the endogenous *Polr2A* gene is fused in-frame with a FLAG tag. Consistent with an interaction, we observed a partial colocalisation of mGFP-tagged FUS and Polr2A, with large patches of FUS granules localising to regions of the nucleus enriched in staining for Polr2A (Fig. 3D).

### FUS is toxic via association with the large subunit of RNA polymerase II

Previous studies have shown that FUS and other FET family members can interact with the large subunit of human RNA polymerase II via its repetitive intrinsically disordered C-terminal domain [28–30]. This interaction between the repetitive tail and FUS has been shown to be length-dependent in vitro [30]. In *Drosophila*, the C-terminal domain consists of 42 degenerate repeats of the consensus heptad YSPTSPS [31]. We reasoned that if interaction between Polr2A and FUS is important for toxicity, then reducing the length of the Polr2A CTD may rescue toxicity by partially blocking this interaction.

**Fig. 4:**
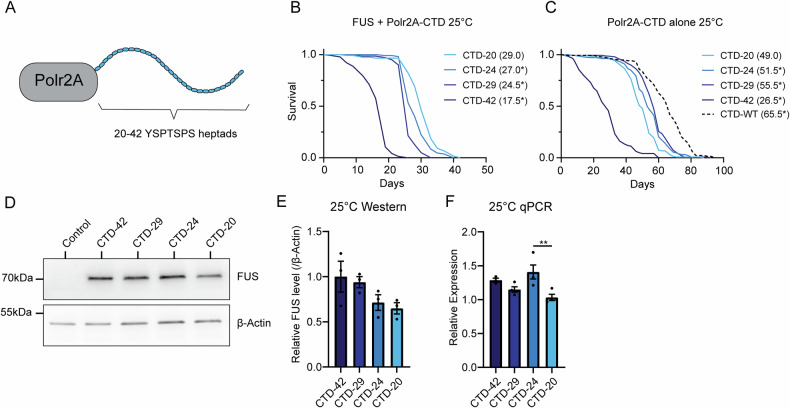
The length of the CTD of Polr2A modifies FUS toxicity in *Drosophila.* **A** Flies with inducible pan-neuronal FUS expression were crossed to lines where the endogenous CTD of Polr2a is replaced with between 20 and 42 consensus YSPTSPS heptad repeats. **B** Lifespans of males expressing FUS pan-neuronally, with expression induced after development at 25 °C. The length of the CTD of Polr2A is indicated. Median lifespan is shown in brackets. *Significant log-rank test in comparison to CTD-20. *n* = 112-142. Full *n* numbers and details of statistical tests are given in Supplementary Tables 3 and 4. **C** Lifespan of flies carrying the modified Polr2A alone at 25 °C. *Significant log-rank test in comparison to CTD-20. *n* = 135–142. Full *n* numbers and details of statistical tests are given in Supplementary Tables 5 and 6. **D** Representative western blot of FUS expression in flies with different CTD lengths when lifespan is performed at 25 °C. Control is a wild-type fly, not expressing human FUS, which demonstrates antibody specificity. **E** Quantification of FUS protein abundance after 6 days at 25 °C. No significant differences in FUS abundance were observed. One way ANOVA (F (3, 8) = 2.613, *P* = 0.123). *n* = 3 independent samples per condition. Bars are mean ± SEM, individual data points are shown. **F** Quantification of FUS transcript expression using qPCR with primers against the N-terminal coding region of FUS. One-way ANOVA (F (3, 11) = 6.458, *P* = 0.0088), **P = 0.0073 Tukey’s multiple comparisons test. Tukey's multiple comparisons tests between all other conditions were non-significant (*P*>0.05). *n*(CTD-42) = 3 *n*(CTD-29), *n*(CTD-24), *n*(CTD-20)=4 independent samples per condition. Bars are mean ± SEM, individual data points are shown.

To assess whether altering the length of the CTD of Polr2A could affect toxicity, we made use of flies where the endogenous *Polr2A* gene had been modified using CRISPR/Cas9 and homology-directed repair to create Polr2A subunits with a CTD composed entirely of the consensus heptad (YSPTSPS) of varying lengths (42, 29, 24, 20 repeats) (Fig. 4A). We crossed together these lines with flies that express FUS pan-neuronally in a temperature-dependent manner. The resulting flies can express FUS and are hemizygous for the indicated X-chromosomal *Polr2A* gene. Because wild-type FUS overexpression is extremely toxic at 29 °C, we assessed toxicity at 25 °C, where expression of FUS is partially repressed. We observed that the lifespan of the FUS-expressing flies was negatively correlated with the repeat length of the large subunit of RNA polymerase II. Flies with the shortest repeat lengths (20 repeats, CTD-20) lived longest (Fig. 4B). This is consistent with the idea that FUS interacts with the CTD to mediate toxicity. However, it remained possible that altering the CTD of Polr2A may reduce or enhance lifespan independently of FUS expression, explaining these differences in lifespan. To test this, we assessed the lifespan of flies carrying the above *Polr2A* variants alone. In addition, we assessed the lifespan of flies with a wild-type *Polr2A* (CTD-WT). All the lines with consensus CTDs of varying lengths had a reduced lifespan compared to flies with a wild-type CTD, suggesting that there has been selection for divergence from the consensus CTD motif (Fig. 4C). Among the flies expressing the consensus CTD at different lengths, we found that 42 repeat-carrying flies had reduced lifespan with or without FUS expression. However, flies with 29 consensus heptads showed the longest lifespan, implying that the reduced toxicity in 24 and 20-repeat expressing flies in the presence of FUS was not simply due to improvement in the fitness of the organism (Fig. 4C). We performed the same experiment at 29 °C to ensure consistency and observed similar results (Supplementary Fig. 3A, B).

Taken together, our results imply that FUS is toxic via an association dependent on the CTD of Polr2A. To rule out that changing the CTD affects the levels of FUS protein itself, we performed immunoblotting for FUS in the different fly lines. Although we observed a trend towards a reduction in FUS abundance with decreasing repeat length, we did not observe significant differences at 25 °C (Fig. 4D, E) but saw more pronounced differences at 29 °C (Supplementary Fig. 3C, D). This implies that differences in toxicity are not primarily driven by reduced FUS abundance. To ensure that any differences were not dependent on reduced transcription of the FUS transgene, we performed qPCR for FUS transcript abundance in the different fly lines. We did not observe any differences that could explain the effect on FUS protein abundance (Fig. 4F and Supplementary Fig. 3E).

### POLR2A mislocalises in patient post-mortem tissue

**Fig. 5:**
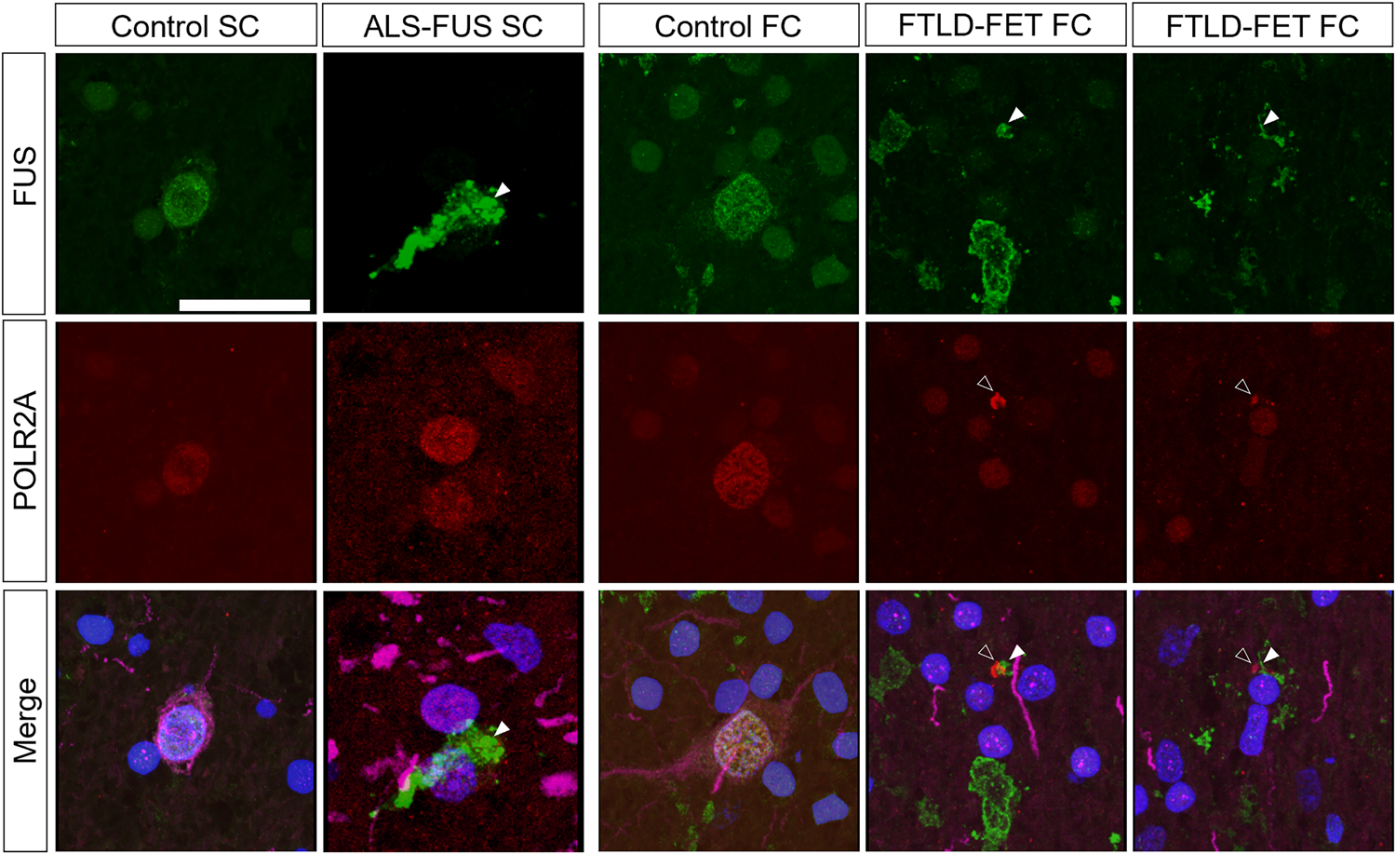
POLR2A inclusions are observed in FTLD-FET but not ALS-FUS human tissue. Tissue sections from patient post-mortem material were stained for FUS as well as human POLR2A. FUS signal is shown in green, POLR2A in red, DAPI in blue and the neuronal marker MAP2 in magenta. FUS inclusions are indicated by white arrowheads, POLR2A inclusions are indicated by black arrowheads. In healthy control spinal cord (SC) and ALS-FUS (R495*) SC POLR2A is predominantly intranuclear, despite the presence of FUS inclusions in the ALS-FUS spinal cord. In the frontal cortex (FC), inclusions of both FUS and POLR2A are observed in the same neuron in FTLD-FET. Two examples are shown. Inclusions of either protein are absent in the control FC. Scale bar = 25 µm.

Our findings suggest that wild-type human FUS can interact with *Drosophila* Polr2A and cause toxicity and that this interaction is suppressed by preventing FUS from entering the nucleus. Given that numerous studies have shown that FUS can interact with human POLR2A, we wondered whether the interaction between FUS and POLR2A may be affected in diseased neurons from patients with FUS-positive proteinopathies. We assessed the distribution of POLR2A and FUS in the spinal cord of two patients with ALS-associated FUS mutations (R495*, K510E), as well as in three healthy controls (details in Supplementary Table 7). Although we saw FUS-positive inclusions in the ALS patients, we observed a normal POLR2A intranuclear distribution in both patients and controls (Fig. 5). In contrast, when we assessed the distribution of POLR2A in the frontal cortex of patients presenting with FTLD-FET, we observed the presence of cytoplasmic puncta of POLR2A in FUS inclusion bearing cells in two out of three cases (example images shown in Fig. 5). These cytoplasmic puncta in some cases partially colocalised with the cytoplasmic FUS inclusions. Inclusions of FUS and POLR2A were absent in healthy control frontal cortex (three cases, example images shown in Fig. 5). These data indicate that the FUS POLR2A interaction may be important both in mediating toxicity in our *Drosophila* models, as well as toxicity in human FTLD-FET patients.

## Discussion

In this study, we have shown that overexpressed human FUS protein is toxic via a nuclear mechanism in *Drosophila* models of FUS-associated ALS/FTLD. FUS forms dynamic structures within the nucleus of *Drosophila* tissue and colocalises with regions of the genome enriched in RNA-polymerase II (Polr2A). Reducing the length of the intrinsically disordered tail of the large subunit of RNA-polymerase II (Polr2A) rescues toxicity in FUS-expressing flies, suggesting that this interaction is important for toxicity. Finally, we observed that in post-mortem material from FTLD-FET, but not ALS-FUS, FUS inclusions coincide with mislocalisation of the large subunit of RNA polymerase II (POLR2A).

Overexpression of wild-type and ALS-mutation bearing human FUS protein, as well as other FET family members, has frequently been used in *Drosophila* in an attempt to develop preclinical models of ALS [21–25, 32–34]. Here, we followed up on our previous observation that deletion of the FUS nuclear localisation sequence prevents motor dysfunction and other neurotoxicity-related phenotypes like reduced lifespan in *Drosophila* [25]. We confirmed that cytoplasmic mislocalisation of human FUS is preferential in terms of toxicity compared to nuclear accumulation. Despite being expressed at similar mRNA levels, the ΔNLS FUS protein is generally present in neurons at lower levels than WT, suggesting that nuclear accumulation may stabilise FUS protein. Despite these differences in levels, ΔNLS FUS accumulates to a greater level in the cytoplasm of neurons than WT FUS, suggesting that FUS is not toxic via a cytoplasmic mechanism. Using variable temperature experiments, we have shown that even when ΔNLS FUS is present at equivalent levels to WT FUS, it is non-toxic. We note that the ΔNLS FUS construct still induced some toxicity when looking at lifespan compared to controls, perhaps suggesting that some mild cytoplasmic toxicity can occur in *Drosophila*.

These results may be somewhat surprising, given that many ALS-associated *FUS* mutations are associated with cytoplasmic mislocalisation of the protein [11–13]. However, in *Drosophila*, overexpressed ALS-mutant human FUS is not typically reported to be particularly more toxic than overexpressed wild-type FUS [23–25] and previously observed differences in toxicity may be due to effects from the transgenic insertion site [21, 34].

Similarly to flies, it has been found that overexpression of wild-type human FUS in the mouse nervous system results in strong toxicity [35, 36]. It has been shown that combining multiple copies of prion-promoter-driven wild-type FUS, resulting in a level 2 fold over non-transgenic animals, is sufficient to drive loss of NMJs and death of the mice after ~4 weeks [37]. Notably, these phenotypes can occur without overt FUS cytoplasmic mislocalisation or aggregation [36, 37], potentially pointing to a nuclear mechanism of toxicity.

Therefore, a key question is whether the nuclear toxicity induced by overexpression of wild-type FUS bears relevance to the disease seen in humans. FUS tightly regulates its own expression and translation [38, 39]. This autoregulatory mechanism suggests that high levels of FUS may be detrimental to cells. Indeed, it has been suggested that failure to autoregulate, in the context of ALS-associated mutations, could drive the disease [39]. Interestingly, it has been reported that point mutations within the FUS 3’UTR in ALS patients result in increased expression of wild-type FUS protein by stabilising the FUS transcript [40, 41]. However, mutations within the 3’UTR have not yet been confirmed to segregate with the disease in patient kindreds. Recent results have also demonstrated that commonly identified FUS mutations can lead to nuclear phenotypes like the accumulation of dysfunctional paraspeckles independently of cytoplasmic localisation or loss-of-function [42]. Furthermore, we and others have identified modifiers of FUS toxicity in *Drosophila* that have similarly affected ALS-associated phenotypes in patient models or tissue[43, 44]. This indicates that there may be some overlap between the nuclear toxicity that we observe in flies and the neurotoxic phenotypes in patients.

There are differences between the FUS aggregates in neurons of individuals with ALS and FTLD. These include whether aggregates consist of mutated versus wild-type FUS, their localisation (both brain region and subcellular localisation), their post-translational modification and their co-aggregation with other proteins [10]. The finding that RNA polymerase II is mislocalised in FUS inclusion-bearing cells exclusively in FTLD-FET but not ALS-FUS further highlights the differences between these two diseases. In support of these differing routes of pathogenesis, a recent cryo-EM study has suggested that the major amyloid-forming protein in FTLD-FET is TAF15 rather than FUS and that FUS is recruited to TAF15 inclusions during the course of the disease [18]. While our study focused on FUS, overexpression of wild-type human TAF15 is similarly toxic to *Drosophila* neurons [32]. Given the strong homology between the two proteins and the fact that TAF15 interacts with the POLR2A CTD as well [30, 45], we suggest that *Drosophila* TAF15 and FUS models are likely toxic via the same mechanism.

The C-terminal region of the large subunit of RNA polymerase (Polr2A) is conserved from yeast to human, consisting of 52 degenerate repeats of the consensus sequence YSPTSPS in humans, while the *Drosophila* Polr2A CTD consists of 42 degenerate repeats [31]. The CTD extends from the core of the enzyme, forming a tail and thus providing binding sites for proteins involved in transcription and RNA processing [46, 47]. The CTD becomes post-translationally modified during the transcriptional cycle, notably undergoing phosphorylation at serine 5 at the transcriptional start site and serine 2 during transcriptional elongation [46]. FET family proteins, including FUS, can interact with POLR2A [27, 45, 48], with several studies implicating a specific interaction with the CTD of POLR2A [28–30]. In vitro hydrogels formed of the recombinant prion-like domain of the FET family proteins are able to trap the recombinant CTD protein in a length-dependent manner [30]. Further studies have shown that the CTD can co-partition into liquid-liquid phase-separated droplets of the FUS protein in vitro and in cells [49, 50]. We have previously found that deletion of domains of FUS required for the formation of hydrogels or liquid-liquid phase separation of FUS protein diminishes toxicity in *Drosophila* models [25], further implicating the interaction between FUS and RNA polymerase II in toxicity.

Previous studies have demonstrated that flies expressing a Polr2A CTD of entirely consensus YSPTSPS heptads are hemizygous viable. However, flies show reduced fitness when the repeat length becomes too long (42 repeats or greater) or too short (20 repeats or fewer) [31]. Our lifespan experiments in otherwise wild-type flies mirror this finding; flies with an intermediate repeat length (29 repeats) were the longest lived. In the context of FUS overexpression, we found that shorter repeat lengths (20 repeats) were preferred, supporting a model where FUS is toxic via association with the Polr2A CTD. We note that we have not yet been able to show a direct interaction between FUS and Polr2A in *Drosophila*, so it remains possible that FUS and Polr2A affect one another indirectly. If FUS does interact with Polr2A directly, an obvious candidate mechanism of toxicity would be disruption of mRNA transcription. This seems particularly plausible in the FTLD patient tissue, where at end-stage, many of the polymerase II positive inclusions are cytoplasmic, hinting at a loss of nuclear function. Consistent with a neuronal sensitivity to inhibited RNA polymerase function, germline mutations that inhibit the function of POLR2A have recently been associated with a range of childhood symptoms, including hypotonia and developmental delay [51]. It is also notable that inhibition of mRNA transcription has been shown to lead to FUS cytoplasmic mislocalisation, suggesting a possible feed-forward loop between transcriptional inhibition and FUS aggregation [52]. Further work will be required to observe whether a transcriptional repression is occurring in FTLD.

In contrast to previous studies [22], we found that FUS does not appear to form insoluble aggregates in our flies. We suggest that the previous reports of insolubility may be dependent on the temperature-sensitive insolubility that we observed. Consistently, we observed that FUS forms granules within the nucleus, with dynamic exchange of FUS between these granules, suggesting that they are not protein aggregates. We note that we primarily observed these granules in (non-neuronal) salivary gland tissue, because these nuclei are very large, facilitating imaging. The non-neuronal nature of these cells is a potential limitation of the current study. Nevertheless, in fixed tissue, we have observed FUS forming granules within the nucleus of *Drosophila* neurons as well. FUS has been described to localise to similar intranuclear granules within human cells [53]. A recent study examining intranuclear blue-light inducible FET family optodroplets suggested that high local concentrations of the POLR2A CTD may be sufficient to nucleate the assembly of FET family proteins into granules [50]. Consistent with the idea that Polr2A may be nucleating the FUS granules that we observed in *Drosophila*, we observed an enrichment of the FUS granules in regions of the nucleus with high levels of Polr2A staining, using flies where the endogenous Polr2A protein had been tagged with a FLAG epitope tag. The FUS granules themselves do not appear to be Polr2A positive, although we had difficulty detecting a strong Polr2A-FLAG signal, even using a modified staining protocol. Future studies looking at the distribution of FUS and Polr2A tagged with fluorescent proteins may be useful to further understand the relationship between the Polr2A CTD and the FUS granules.

In this study, we have assessed a potential role for POLR2A in ALS-FUS and FTLD-FET. We have assessed tissue from a small number of patients and found evidence that POLR2A may become mislocalised in FTLD-FET but not ALS-FUS. We also note that inclusions of the large subunit of RNA polymerase II have recently been found to colocalise with TDP-43 inclusions in FTLD [54], as well as occurring in Tau inclusion-bearing cells in Alzheimer’s disease [55], suggesting that RNA polymerase II dysfunction could be a common mechanism in multiple forms of FTLD. We note that a limitation of our study is the limited number of cases assessed. Future replication studies assessing a large number of patients of different FTLD subtypes are therefore warranted.

In conclusion, overexpression of the human FUS protein is toxic in animal models. However, in *Drosophila,* cytoplasmic accumulation of FUS is less detrimental than nuclear accumulation. Within the nucleus, FUS forms toxic granules in association with RNA polymerase II, in a manner dependent on interaction with its intrinsically disordered CTD. This association with RNA polymerase II may be relevant to human disease, as we observe accumulation of POLR2A aggregates in the brains of patients with FTLD-FET.

## Materials and methods

### Drosophila

The following stocks were obtained from the Bloomington *Drosophila* stock centre: w1118 (5905), D42-GAL4 (8816), tubP-GAL80^ts^ (7019), nSyb-GAL4 (51635), fkh-GAL4 (78060). w^-^; dilp2-GAL4, UAS-CD8-GFP/ CyO; btl-GAL80 was obtained from Patrick Callaerts (KU Leuven, Belgium). The UAS-FUS WT and UAS-FUS ΔNLS lines inserted at VK00031 landing site were described previously [25]. The Polr2A^42con.FLAG^, Polr2A^29con.FLAG^, Polr2A^24con.FLAG^, Polr2A^20con.FLAG^, Polr2A^FLAG^ stocks were previously described [31]. Unless otherwise stated, flies were kept at 25 °C on a 12 h light/dark cycle and males were used in experiments. Full genotypes are given in the supplementary methods. Experiments were performed on standard *Drosophila* media (either: 62.5 g/L cornmeal, 25 g/L yeast, 7 g/L agar, 16.9 g/L dextrose, 37.5 mL/L golden syrup, 9.375 mL/L propionic acid, 1.4 g/L methyl 4-hydroxybenzoate and 14.0 mL/L ethanol or: 10 g/L agar, 5 g/L soya flour, 15 g/L sucrose, 33 g/L glucose, 15 g/L maize meal, 10 g/L wheat germ, 30 g/L molasses, 35 g/L yeast, 5 mL/L priopionic acid, 0.25 g/L methyl 4-hydroxybenzoate, 9 mL/L ethanol).

### Cloning and *Drosophila* transgenesis

DNA encoding wild-type human FUS protein [56] was subcloned into the pUAST attB vector. Note: due to the presence of a 6 bp restriction site to facilitate cloning, the final length of the FUS protein is 528 amino acids. For mGFP tagged constructs: the coding sequence of the *Drosophila* codon optimised mEGFP sequence (mutant A206K) was ordered from IDT as a gBlock. This was subcloned into Pac5.1. The mGFP coding sequence was PCR amplified from this vector with overhangs and Gibson assembled (NEBuilder® HiFi DNA Assembly Cloning Kit) into the linearised FUS pUAST attb vector. Vectors were injected into M{vas-int.Dm}ZH-2A; P{CaryP}attP40 or M{vas-int.Dm}ZH-2A;; P{CaryP}attP2 by BestGene. Resultant transgenics were crossed to w1118 for two generations to remove the X chromosomal integrase.

### Lifespan

Approximately 50 virgins were allowed to mate with males and eggs were collected on grape-agar plates supplemented with yeast paste over a 24 h period, washed with PBS and deposited into bottles of fly food at a standard density. Crosses were allowed to develop at 18 °C for 21 days. On day 21, males were split into vials at a density of 15 flies per vial, with approximately 10 vials per condition (150 flies total). Vials were placed at the indicated temperature and deaths were scored every 1–3 days. Flies were tipped onto fresh food every 2–3 days. Flies that were accidentally killed or escaped during transfer were censored from the data. Data are presented as cumulative survival curves and statistical comparisons are made using the log-rank test. Statistical tests were performed using Microsoft Excel (template available at http://piperlab.org/resources). Uncorrected P-values are reported. Bonferroni correction for multiple comparisons was evaluated but did not change the significance of any comparisons.

### Eclosion assays

5 virgins per vial were crossed to the indicated males. Flies were allowed to mate before laying on food for 48 h. Vials were aged at 25 °C or 29 °C and were scored 12 days after removal of adults. Percentage eclosion was calculated by counting the number of empty pupal cases relative to the total number of pupal cases. Counting was performed blinded and vials with fewer than 20 pupae were excluded a priori from analysis.

### qPCR

Flies were set up as described for lifespan assays at standard density, before males were split into vials and the expression induced by raising the temperature to 25 °C or 29 °C. Three days (29 °C) or 7 days (25 °C) after induction, flies were snap frozen in liquid nitrogen. Total RNA was extracted from 10 heads or per sample using TRI Reagent (Sigma) following the manufacturer’s protocol. One µg of RNA was used per cDNA synthesis reaction using the SuperScript™ III First-Strand Synthesis System (Thermo-Fisher) using random hexamers following the manufacturer’s protocol. qPCR reactions were performed using Fast SYBR Green Master Mix (Applied Biosystems) using the QuantStudio™ 3 Real-Time PCR System (Applied Biosystems). qPCR was performed using primers targeting the QGSY domain of FUS and normalised to the expression of Tubulin 84B and RpL9. The stability of the loading controls was assessed and normalisation performed using qbase+ software (Biogazelle).

Primers were: FUS-For: ggttatagccagtccacgga; FUS-rev: ggggagttgactgagttcca; Tub84B-For: tgggcccgtctggaccacaa; Tub84B-Rev: tcgccgtcaccggagtccat; RpL9-For: catgatcaagggagtcacgt; RpL9-Rev: atgtacttctcacccaagaag.

### Preparation of total protein lysate for western blotting

For adult samples: Flies were allowed to develop at 18 °C before males were split into vials and expression induced at 29 °C for 3 days or 25 °C for 6 days. Flies were snap frozen in liquid nitrogen. Heads were removed and 10 heads per sample were homogenised in 10 µl per head of 2x Lane Marker Reducing Sample Buffer (Pierce) before being boiled at 95 °C for 10 min. Samples were centrifuged at 16,000 x *g* for 10 min and the supernatant was moved to a new tube.

For larval samples: The central nervous systems of L3-stage larvae were dissected in PBS. Five larvae per replicate were dissected. The tissue was centrifuged at 500 x *g* for 5 min and PBS was replaced with 50 µl 1× Lane Marker Reducing Sample Buffer (Pierce). The sample was boiled at 95 °C for 5 min. Samples were centrifuged at 16,000 x g for 10 min and the supernatant was moved to a new tube.

### Western blotting

Samples were either loaded onto 4–12% Novex Bis-Tris gels (Invitrogen) and SDS-PAGE was performed using 1X MOPS buffer (Invitrogen) (Fig. 2B and Supplementary Fig. 2F); or samples were loaded onto Mini-PROTEAN® TGX™ Precast Gels (BioRad) and SDS-PAGE performed using 1X Tris/Glycine/SDS running buffer (BioRad) (Fig. 4D and Supplementary Fig. 3C); or samples were loaded onto 4–12% Criterion™ XT Bis-Tris Protein Gel, 18 well, 30 µl (Biorad) gels and SDS-PAGE performed using 1x MOPS buffer (Biorad) (Fig. 2G). Protein was transferred to a nitrocellulose membrane (BioRad) using the Trans-Blot Turbo transfer system (BioRad). The membrane was blocked for 1 h in 5% skimmed milk in TBST at room temperature. Primary antibodies were applied in TBST overnight at 4 °C or for 1 h at room temperature. Primary antibodies were: anti-N-terminal FUS (Abcam, ab243880) at 1/1000 (Fig. 2B, G); anti-C-terminal FUS (Santa Cruz, sc-47711) at 1/1000 (Fig. 4D and Supplementary Figs. 2; 3); anti beta-actin (Abcam, ab8224) at 1/10,000; anti-histone H4 (Abcam ab10158) at 1/2000; anti alpha-Tubulin (Sigma, T6199) at 1/5000.

HRP-conjugated anti-rabbit or anti-mouse antibodies (Dako: P0447, P0448; or Cell Signalling Technology: 7074S, 7076S) were applied for 1 h at 1/5000 in TBST. Blots were developed using SuperSignal™ Chemiluminescent Substrates (Thermo Fisher) and digitally imaged using an ImageQuant LAS 4000 or Biorad ChemiDoc MP (Biorad) imaging system.

### Immunofluorescence of FUS in larval motor neurons

FUS was expressed using the D42-GAL4 driver line. Crosses were reared at 25 °C. The central nervous system (CNS) of larvae was dissected in PBS and fixed in 4% PFA in PBS for 30 min at room temperature. Tissue was washed 3 times for 10 min in PBST (PBS with 0.3% Triton X-100), before being blocked for 1 h at room temperature in PBST with 10% BSA. Primary antibodies were applied for 48 h in PBST + 10% BSA at 4 °C. Primary antibody was anti-N-terminal FUS (Abcam, ab243880) at 1/300. Following primary incubation, CNSs were washed three times for 10 min in PBST. Secondary antibody, Alexa Fluor™ 488 Donkey anti-rabbit 1/300 (Thermo Fisher, A-21206), and Alexa Fluor™ 647 phalloidin (Cell Signalling Technology, 8940S) 1/100 were applied in PBST + 10% BSA for 2 h at room temperature. CNSs were washed three times for 10 min in PBST, once in PBS and mounted in SlowFade™ Gold Antifade Mountant with DAPI (Thermo Fisher, S36939). Confocal images (16 bit) were obtained with a Zeiss LSM 710 confocal microscope using a Plan-Apochromat 63x/1.40 Oil DIC M27 objective. Images were analyzed in ImageJ. For individual cells, the region of the nucleus was identified using the DAPI channel and an ROI corresponding to the nucleus was generated. The phalloidin channel was used to define an ROI corresponding to the edge of the cell. The raw integrated density of the FUS signal in the whole cell, nucleus and cytoplasm (whole cell–nucleus) was calculated. These values were normalized to the area of each compartment in µm^2^. Nuclear/Cytoplasmic ratios were calculated by taking the integrated density of pixel values in (FUS) 488-channel in the nucleus, calculating the integrated density of pixel values in the cytoplasm and expressing these numbers as a ratio.

#### FLAG-Polr2A immunostaining

Crosses were raised at room temperature (21 °C) until reaching wandering L3 larval stages. Male mGFP-FUS expressing wandering L3 larvae were dissected in room-temperature PBS. Salivary glands were fixed in ice-cold 100% methanol at –20 °C for 20 min. Glands were washed three times for 10 min in PBS + 0.1% Tween-20 (PBST), before being blocked for 10% BSA in PBST for 1 h at room temperature. Glands were incubated in primary anti-FLAG antibody (Sigma, F1804) at 1/300 in block overnight at 4 °C. The following day, samples were washed in PBST 3 times for 10 min. Samples were put into Alexa-555 labelled secondary antibody (A-31570, ThermoFisher) at 1/300 in block for 2 h at room temperature. Samples were washed three times in PBST and mounted in VECTASHIELD® Antifade Mounting Medium with DAPI (Vector Laboratories). Samples were imaged using an inverted confocal microscope (SP8 DMi8, Leica Microsystems) using an HC PL APO CS2 63x/1.40NA oil objective. Images were collected as a Z-stack and are presented as a maximum intensity projection.

### Ex vivo live salivary gland imaging

Crosses were raised at room temperature (21 °C) until reaching wandering L3 larval stages. Larvae were washed in room temperature Schneider’s *Drosophila* Media (Gibco) and the salivary glands dissected in Schneider’s Drosophila Media (Gibco). Salivary glands were placed onto the surface of an ibiTreat-coated 35 mm µ-dish (Ibidi) and covered with a layer of room temperature Schneider’s *Drosophila* Media (Gibco). Images were acquired at room temperature. Images were acquired every 1.02 s using a Nikon TiE A1R confocal microscope using a Plan Apo VC 60x Oil 1.4NA objective and 488 nm laser. For FRAP experiments, a circular stimulation ROI of area 28.94 μm^2^ was used. Two reference ROIs (11.18 μm^2^) were added, one positioned within a region of GFP signal in an adjacent nucleus and one positioned in a GFP-negative region of tissue. During photobleaching, the laser was set to maximum intensity for 2 imaging cycles. Fluorescence intensity in the stimulation ROI was adjusted using the background and reference ROI signal and recovery half-time was calculated using NIS Elements software (Nikon). Images were pseudocoloured using the FIRE look-up table in ImageJ. Timestamps on videos are in seconds.

### Patient tissue

Cases were sourced from the London Neurodegenerative Diseases Brain Bank (King’s College London, U.K.) (Details in Supplementary Table 7). Sections (7 μM) were prepared from paraffin-embedded tissue blocks (10% formalin-fixed).

### Immunofluorescence of patient tissue

Slides underwent washes in de-waxing xylene tanks (2×, 2 min each), 99% Industrial Methylated Spirit (IMS) (2×, 2 min each), 95% IMS (1×, 2 min). Samples were then rinsed under water before being moved to distilled water. Slides then underwent a microwave-enhanced antigen retrieval protocol. Briefly, samples were incubated with Citrate Buffer (0.01 M, pH 6.0) and microwaved at the ‘high’ setting for 6 min, once, then at the ‘simmer’ setting for 8 min, two times. After being washed under running water, slides were washed twice for 5 min in Phosphate Buffered Saline (PBS) and were then incubated in blocking solution (Normal Goat Serum, ab7481, Abcam, 1:10 dilution in PBS) for 45 min. Samples were then incubated in primary antibodies for 1 h at 37 °C. Primary antibodies were: mouse anti-POLR2A, Santa Cruz Biotechnology (sc-47701) at 1:100 dilution; rabbit anti-FUS, Atlas Antibodies (HPA008784) at 1:400 dilution; chicken anti-MAP2, Abcam (ab5392), at 1:5000 dilution. Samples underwent two 5 min washes in PBS (1X) before being incubated in Alexa Fluor-conjugated secondary antibodies for another 45 min at room temperature. Secondary antibodies were: Alexa 488 goat anti-rabbit, Abcam (ab150077) at 1:1000 dilution; Alexa 568 goat anti-mouse, Abcam (ab175473) at 1:1000 dilution; Alexa 647 goat anti-chicken, Abcam (ab150171) at 1:1000 dilution. Two additional washes in PBS (1X) were performed for 5 min each before slides were incubated with a 1X quenching buffer solution (True Black Plus Lipofuscin Autofluorescence Quencher, 40X in DMSO, 23014, Biotium); this step was carried out for 8 min at room temperature. Two more washes for 5 min each were then followed by an incubation step with DAPI for 10 min at room temperature. Sides were then mounted with 2/3 drops of mounting medium (EverBrite Hardset Mounting Medium, 23003, Biotium) using 1.5H glass coverslips (VWR). Immunofluorescent post-mortem spinal cord and prefrontal cortex sections were imaged on a Nikon Upright Ni-E with A1R confocal optics, through a 60x oil 1.4NA objective.

### Statistics

Statistical comparisons were performed using GraphPad Prism or Microsoft Excel. No a priori statistical tests were used to determine sample size. The F-test (two samples) or Brown-Forsythe test (more than two samples) were used to assess homogeneity of variance. Normality was tested using the Shapiro-Wilk test. If the data were non-normally distributed, appropriate non-parametric tests were employed (Mann-Whitney or Kruskal–Wallis tests). If the data were normally distributed but the assumption of homogeneity of variance was not met, then tests robust to unequal variances were employed (Welch’s t-test). Full details of all statistical tests are provided in the Figure legends and Supplementary Tables 1–6 and 8–11. Experiments were not performed blinded unless otherwise stated.

## Supplementary information

Electronic Supplementary Material

Supplementary Movie 1

Supplementary Movie 2

Supplementary Movie 3

Uncropped Western Blots

## Data Availability

The datasets generated during and/or analysed during the current study are available from the corresponding author on reasonable request.
