# Supplementary material for: Human FUS is toxic via association with RNA polymerase II in Drosophila
Source: Cell Death Dis. 2026 Mar 14;17(1):310. doi: 10.1038/s41419-026-08539-x (PMC13039543; doi:10.1038/s41419-026-08539-x)
Supplement: Supplementary file 1 — Electronic Supplementary Material [file 41419_2026_8539_MOESM1_ESM.pdf]

Electronic Supplementary Material
Human FUS is toxic via association with RNA polymerase II in *Drosophila*

Thomas G. Moens[1,2,3*], Luca Biasetti[4], Wendy Scheveneels[1,2], Bradley N. Smith[4], Claire Troakes[4], Philip Van Damme[1,2,5], Caroline Vance[4], Ludo Van Den Bosch[1,2,*]

1. KU Leuven – University of Leuven, Department of Neurosciences, Experimental Neurology and Leuven Brain Institute (LBI), Leuven, Belgium.

2. VIB, Center for Brain & Disease Research, Laboratory of Neurobiology, Leuven, Belgium.

3. Current Address: CRUK Scotland Institute, Garscube Estate, Switchback Road, Glasgow, G61 1BD, UK.

4. Maurice Wohl Clinical Neuroscience Institute and the Institute of Psychiatry, Psychology and Neuroscience, King's College London, Camberwell, London, SE5 9RT, UK

5. University Hospitals Leuven, Department of Neurology, Leuven, Belgium

*Corresponding Authors: Email: ludo.vandenbosch@kuleuven.be, t.moens@crukscotlandinstitute.ac.uk

Supplementary Materials and Methods

Full *Drosophila* Genotypes

Figure 1: B) Control = w^- ;; D42-GAL4 /+, FUS WT = w^- ;; UAS-FUS WT / D42-GAL4, FUS ΔNLS = w^- ;; UAS-FUS ΔNLS / D42-GAL4. **C)** Control= w^- ;;nSyb-GAL4 / +, FUS WT = w^- ;; UAS-FUS WT / nSyb-GAL4, FUS ΔNLS = w^- ;; UAS-FUS ΔNLS / nSyb-GAL4 **D-F)** Control = w^- ;; D42-GAL4 /+, FUS WT = w^- ;; UAS-FUS WT / D42-GAL4, FUS ΔNLS = w^- ;; UAS-FUS ΔNLS / D42-GAL4.

Figure 2: A-D) Control = w^- ; tubP-GAL80^{ts} /+ ; nSyb-GAL4 / +, FUS WT = w^- ; tubP-GAL80^{ts} / + ; UAS-FUS WT / nSyb-GAL4, FUS ΔNLS = w^- ; tubP-GAL80^{ts} /+ ; UAS-FUS ΔNLS / nSyb-GAL4. **E-H)** Control = w^- ;; D42-GAL4 /+, FUS WT = w^- ;; UAS-FUS WT / D42-GAL4, FUS ΔNLS = w^- ;; UAS-FUS ΔNLS / D42-GAL4.

Figure 3: mGFP-FUS = w$^-$; + ; UAS-mGFP-FUS / fkh-GAL4, mGFP-NLS = w$^-$; + ; UAS-mGFP-NLS / fkh-GAL4.

Figure 4: B,D,E,F) CTD-42 = Polr2A$^{42con.FLAG}$, w$^-$ / Y; tubP-GAL80^{ts}, UAS-FUS WT / + ; nSyb-GAL4 / +, CTD-29 = Polr2A$^{29con.FLAG}$, w$^-$ / Y; tubP-GAL80^{ts}, UAS-FUS WT / + ; nSyb-GAL4 /+, CTD-24 = Polr2A$^{24con.FLAG}$, w$^-$ / Y; tubP-GAL80^{ts}, UAS-FUS WT / + ; nSyb-GAL4 / +, CTD-20 = Polr2A$^{20con.FLAG}$, w$^-$ / Y; tubP-GAL80^{ts}, UAS-FUS WT / + ; nSyb-GAL4 / +. Control = w1118 (w- ; + ; +). **C)** CTD-42 = Polr2A$^{42con.FLAG}$, w$^-$ / Y; + ; + , CTD-29 = Polr2A$^{29con.FLAG}$, w$^-$ / Y; + ; + , CTD-24 = Polr2A$^{24con.FLAG}$, w$^-$ / Y ; +; + , CTD-20 = Polr2A$^{20con.FLAG}$, w$^-$ / Y; + ; + , CTD-WT = Polr2A^{FLAG}, w$^-$ / Y; + ; +.

Supplementary Figure 1: A-C) Control = w$^-$; dilp2-GAL4, UAS-cd8-GFP/ tubP-GAL80^{ts}; btl-GAL80 /+, FUS WT = w$^-$; dilp2-GAL4, UAS-cd8-GFP/ tubP-GAL80^{ts}; btl-GAL80 / UAS-FUS WT, FUS ΔNLS= w$^-$; dilp2-GAL4, UAS-cd8-GFP/ tubP-GAL80^{ts}; btl-GAL80 / UAS-FUS ΔNLS.

Supplementary Figure 2: A) mGFP-FUS = w$^-$;; fkh-GAL4 / UAS-mGFP-FUS, mGFP-NLS = w$^-$;; fkh-GAL4 / UAS-mGFP-NLS. **B)** mGFP-FUS = w$^-$;; D42-GAL4 / UAS-mGFP-FUS, mGFP-NLS = w$^-$;; D42-GAL4 / UAS-mGFP-NLS. **C)** Control = w$^-$;; fkh-GAL4 /+, FUS= w$^-$; UAS-FUS/+ ; fkh-GAL4 / +. **D)** mGFP-FUS = w$^-$; tubP-GAL80^{ts} / + ; UAS-mGFP-FUS / nSyb-GAL4, mGFP-NLS = w$^-$; tubP-GAL80^{ts} / + ; UAS-mGFP-NLS / nSyb-GAL4. **E)** mGFP-FUS = w$^-$;; fkh-GAL4 / UAS-mGFP-FUS, mGFP-NLS = w$^-$;; fkh-GAL4 / UAS-mGFP-NLS. **F)** w$^-$; tubP-GAL80^{ts} / + ; UAS-FUS / nSyb-GAL4.

Supplementary Figure 3: A,C,D,E) CTD-42 = Polr2A$^{42con.FLAG}$, w$^-$ /Y; tubP-GAL80^{ts}, UAS-FUS WT/+ ; nSyb-GAL4 /+, CTD-29 = Polr2A$^{29con.FLAG}$, w$^-$ /Y; tubP-GAL80^{ts}, UAS-FUS WT/+ ; nSyb-GAL4 /+, CTD-24 = Polr2A$^{24con.FLAG}$, w$^-$/Y; tubP-GAL80^{ts}, UAS-FUS WT/+ ; nSyb-GAL4 /+, CTD-20 = Polr2A$^{20con.FLAG}$, w$^-$ /Y; tubP-GAL80^{ts}, UAS-FUS WT/+ ; nSyb-GAL4 /+. Control = w1118 (w- ; + ; +). **B)** CTD-42 = Polr2A$^{42con.FLAG}$, w$^-$ /Y; + ; + , CTD-29 = Polr2A$^{29con.FLAG}$, w$^-$/Y;

+ ; +, CTD-24 = Polr2A$^{24con.FLAG}$, w^{-} / Y; + ; +, CTD-20 = Polr2A$^{20con.FLAG}$, w^{-} / Y; + ; +, CTD-WT = Polr2A^{FLAG}, w^{-}/Y; + ; +.

Temperature dependent RIPA fractionation

Flies were allowed to develop at 18°C before males were split into vials and expression induced at 29°C for 3 days. Flies were anaesthetized on CO_2 and heads removed using dissection scissors. 10 heads were either collected into an Eppendorf tube on ice or at room temperature and were homogenized using a Kontes pellet pestle in 110µl RIPA buffer (Sigma, 150 mM NaCl, 1.0% IGEPAL® CA-630, 0.5% sodium deoxycholate, 0.1% SDS, 50 mM Tris, pH 8.0) supplemented with 1X Roche cOmplete™ EDTA-free Protease Inhibitor Cocktail. The RIPA buffer was either ice-cold or kept at room temperature prior to use. Once heads were homogenised lysis was allowed to proceed for 20 minutes on ice or at room temperature. The samples were centrifuged at 16,000 x g for 10 minutes at either 4°C or room temperature. 100µl of supernatant was recovered and combined with 25µl of 5X Lane Marker Reducing Sample Buffer (Pierce) before being boiled at 95°C for 5 minutes. This was kept as the soluble fraction. The pellet from the centrifugation step was washed in 100µl of room temperature or ice cold RIPA buffer before being re-centrifuged in the same manner. The supernatant was removed and discarded and the pellet resuspended in 125µl 1X Lane Marker Reducing Sample Buffer (Pierce) before being boiled at 95°C for 5 minutes. To remove residual insoluble material, the resuspended pellet was centrifuged at 16,000xg for 5 minutes at room temperature. The supernatant was retained as the insoluble protein fraction.

Immunofluorescence of FUS in Insulin-producing neurons

FUS was expressed in insulin-producing neurons by recombining UAS-FUS lines with tubP-GAL80^{ts} and crossing these lines to males of w-; dilp2-GAL4, uas-cd8-GFP/ CyO; btl-GAL80. Note: btl-gal80 suppresses expression in the trachea during development[1]. Crosses were allowed to develop at 18°C and adults allowed to eclose over a 24 h period. Adult males of

the indicated genotype (see Full *Drosophila* Genotypes) were split into vials and expression induced at 29°C for 5 days. Brains were dissected in cold PBS and fixed in 3.7% formaldehyde (Sigma), in PBST (0.3% Triton X-100) for 20 min at room temperature. Brains were washed 3 times in PBST before being blocked for 1 h at room temperature in PBST with 10% BSA. Primary antibody was applied for 48 h in PBST + 10% BSA at 4°C. Primary antibody was anti-N-terminal FUS (BD Biosciences 611384) at 1/500. Following primary incubation, brains were washed 3 times for 10 min in PBST and secondary antibody, Alexa Fluor™ 555 Donkey anti-Mouse (Thermo Fisher, A-31570) was applied at 1/500 in PBST + 10% BSA for 2 h at room temperature. Brains were washed 3 times for 10 min in PBST, once more in PBS and mounted in vectashield with DAPI (Vectorlabs, H-1200). Confocal images (8 bit) were obtained with Leica SP8 DMI8 confocal microscope using an HC PL APO CS2 63x/1.40 NA Oil objective and images were analyzed in ImageJ. The region of the nucleus and the cell body was identified using the GFP channel, and an ROI corresponding to the nucleus and cell body generated. Nuclear/Cytoplasmic ratios were calculated by taking the integrated density of pixel values in 555-channel in the nucleus, calculating the integrated density of pixel values in the cytoplasm (whole cell minus nucleus) and expressing these numbers as a ratio.

mGFP-FUS brain imaging

Flies carrying the indicated mGFP-FUS or mGFP-NLS construct were crossed to flies carrying tubP-GAL80^{ts} and nSyb-GAL4. Flies were allowed to develop at 18°C before males were split into vials and expression induced at 29°C for 3 days. Brains were dissected in room temperature PBS, fixed in 3.7% PFA in PBS for 20 minutes at room temperature, and washed two further times in PBS. Samples were mounted in VECTASHIELD® Antifade Mounting Medium with DAPI (Vector Laboratories). Samples were imaged using an inverted Leica SP8 DMI8 confocal microscope using an HC PL APO CS2 63x/1.40 NA Oil objective. Images were taken of nuclei ventral to the antennal lobe.

Salivary gland dissection and imaging

Quantification of FUS-GFP signal intensity: Crosses were raised at room temperature (21°C) until reaching wandering L3 larval stages. Wandering L3 larvae were dissected in room-temperature PBS. Salivary glands were fixed in 3.7% PFA (Sigma) in PBS for 20 minutes at room temperature. **Untagged FUS immunostaining:** Crosses were raised at room temperature (21°C) until reaching wandering L3 larval stages. Wandering L3 larvae were dissected in room-temperature PBS. Salivary glands were fixed in 3.7% PFA (Sigma) in PBS for 20 minutes at room temperature. Tissue was washed 3 times in PBST (0.3% Triton X-100) before being blocked for 1 hour at room temperature in PBST with 10% BSA. Primary antibodies were applied for 48 hours in PBST + 10% BSA at 4°C. The tissue was washed 3 times for 10 minutes in PBST and secondary antibody, in PBST + 10% BSA for 2 hours at room temperature. Brains were washed 3 times for 10 minutes in PBST, once in PBS before mounting. Primary antibody was anti-N-terminal FUS (BD Biosciences 611384) at 1/300. Secondary antibody was Alexa Fluor™ 555 Donkey anti-Mouse (Thermo Fisher, A-31570) used at 1/300. **Oligo-dT FISH:** Crosses were raised at room temperature (21°C) until reaching wandering L3 larval stages. Wandering L3 larvae were dissected in PBS and tissue fixed in 3.7% PFA in PBS for 20 minutes at room temperature. Samples were washed in PBS and moved to ice cold 100% methanol for 20 minutes at -20°C. Samples were stored in 70% ethanol at -20°C until use. Samples were washed for 5 minutes in 1M Tris.HCl (pH 8.0) for 5 minutes. The tissue was then incubated in hybridisation buffer: 2X SSC (Fisher BioReagents), 1mg/mL Yeast tRNA (AM7119, ThermoFisher), 0.005% BSA (B8667, Sigma), 10% Dextra sulfate (D8906, Sigma), 25% deionized formamide (Sigma) made in RNAse-free H_2O, supplemented with 1ng/µl 5’Cy3-labelled-dT_{30} DNA probe (IDT) at 37°C overnight. The following day the samples were washed once for 10 minutes with 4X SCC, and three times with 2X SSC.

Samples were mounted in VECTASHIELD® Antifade Mounting Medium with DAPI (Vector Laboratories). Samples were imaged using an inverted confocal microscope (SP8 DMi8, Leica Microsystems) using an HC PL APO CS2 63x/1.40 NA Oil objective.

Supplementary Figure 1

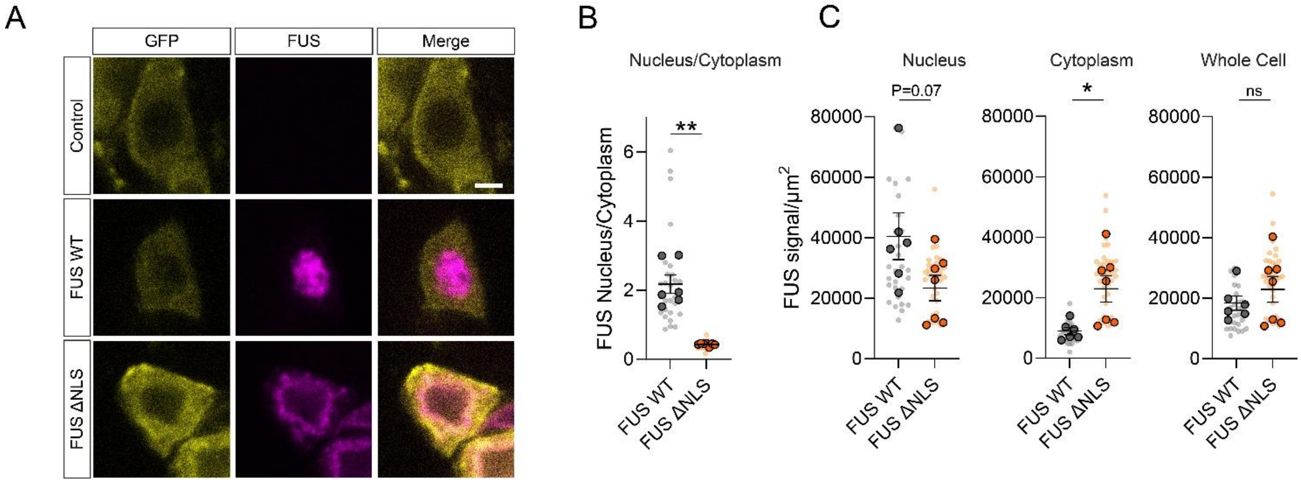

Supplementary Figure 1, related to Figure 2. A) Confocal microscopy images of insulin-producing neurons in the adult fly brain expressing FUS constructs along with cd8-GFP. FUS was detected using an N-terminal monoclonal antibody. Control flies expressed GFP alone. Scale bar = 3µm. **B)** Quantification of the ratio of nuclear/cytoplasmic FUS protein in FUS WT and FUS ΔNLS expressing insulin-producing neurons. Plots show values from individual cells (small data points) as well as the average from each brain (large data points). Number of individual neurons analysed: (WT)=28, n(ΔNLS)=36. Number of individual brains analysed: n(WT)=6, n(ΔNLS)=7. **P=0.0012, two-tailed Mann-Whitney test comparing brain averages. Bars are mean ± SEM of brain averages. **C)** Plots showing the FUS signal intensity normalised to area (μm^2) for the nucleus, cytoplasm and whole-cell. Plots show values from individual cells (small data points) as well as the average from each brain (large data points). Number of individual neurons analysed: (WT)=28, n(ΔNLS)=36. Number of individual brains analysed: n(WT)=6, n(ΔNLS)=7. P value nucleus = 0.0684, two-tailed t-test comparing brain averages. P value cytoplasm = *0.0179, Welch's two-tailed t-test comparing brain averages. P value whole cell = 0.3930, two-tailed t-test comparing brain averages. ns=non-significant. Bars are mean ± SEM of brain averages.

Supplementary Figure 2

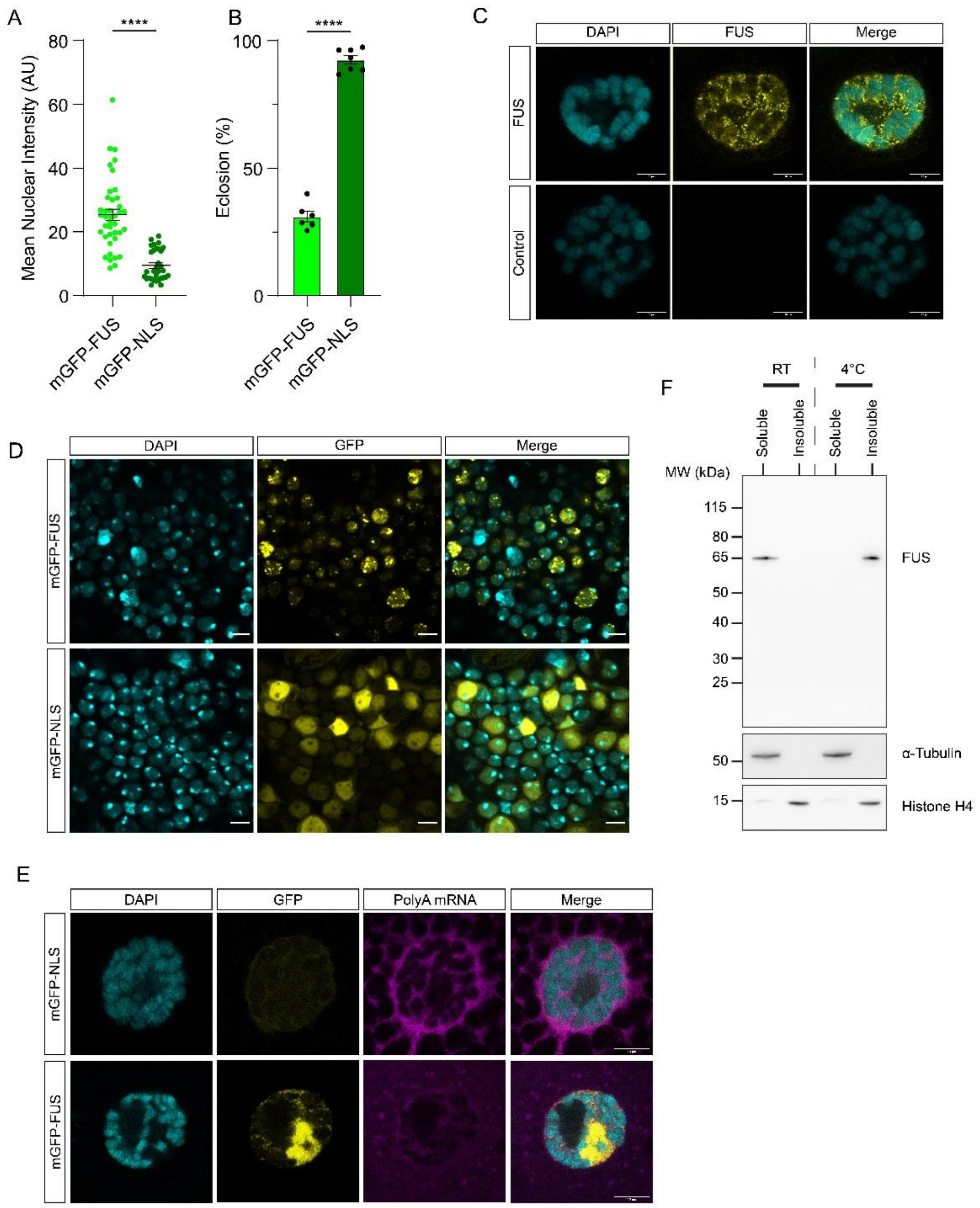

Supplementary Figure 2, related to Figure 3. A) The nuclear intensity of mGFP signal was assessed in salivary gland nuclei expressing mGFP-FUS or mGFP-NLS. ****P<0.0001, two-tailed Mann-Whitney test. n=32-40 nuclei from 5 larvae, bars are mean ± SEM, individual data points are shown. **B)** mGFP-FUS and mGFP-NLS flies were crossed to D42-GAL4 at 25°C and eclosion assessed. ****P<0.0001 two-tailed T-test. n=6-7 vials, bars are mean ± SEM, individual data points are shown. **C)** Salivary glands expressing untagged wild-type FUS protein were immunostained for FUS. Driver alone (fkh-GAL4 / +) was used as a control. No FUS signal was observed in the control, confirming antibody specificity. Scale bar = 10µm **D)** Expression of mGFP-FUS or mGFP-NLS was driven in adult neurons using the nSyb-GAL4 driver. A punctate intranuclear distribution of mGFP-FUS but not mGFP-NLS was observed. Scale bar = 5µm. **E)** FISH on mGFP-FUS or mGFP-NLS expressing salivary glands was performed to label polyA mRNA. mGFP-FUS positive puncta were not overtly mRNA positive. Scale bar = 10µm **F)** Fractionation of heads of flies expressing FUS pan-neuronally was performed either at room temperature (RT) or on ice (4 ºC). FUS is predominantly soluble when extracted in room temperature RIPA buffer but is insoluble when extracted at 4 ºC. α-Tubulin and Histone H4 serve as RIPA soluble and insoluble positive controls respectively.

Supplementary Figure 3

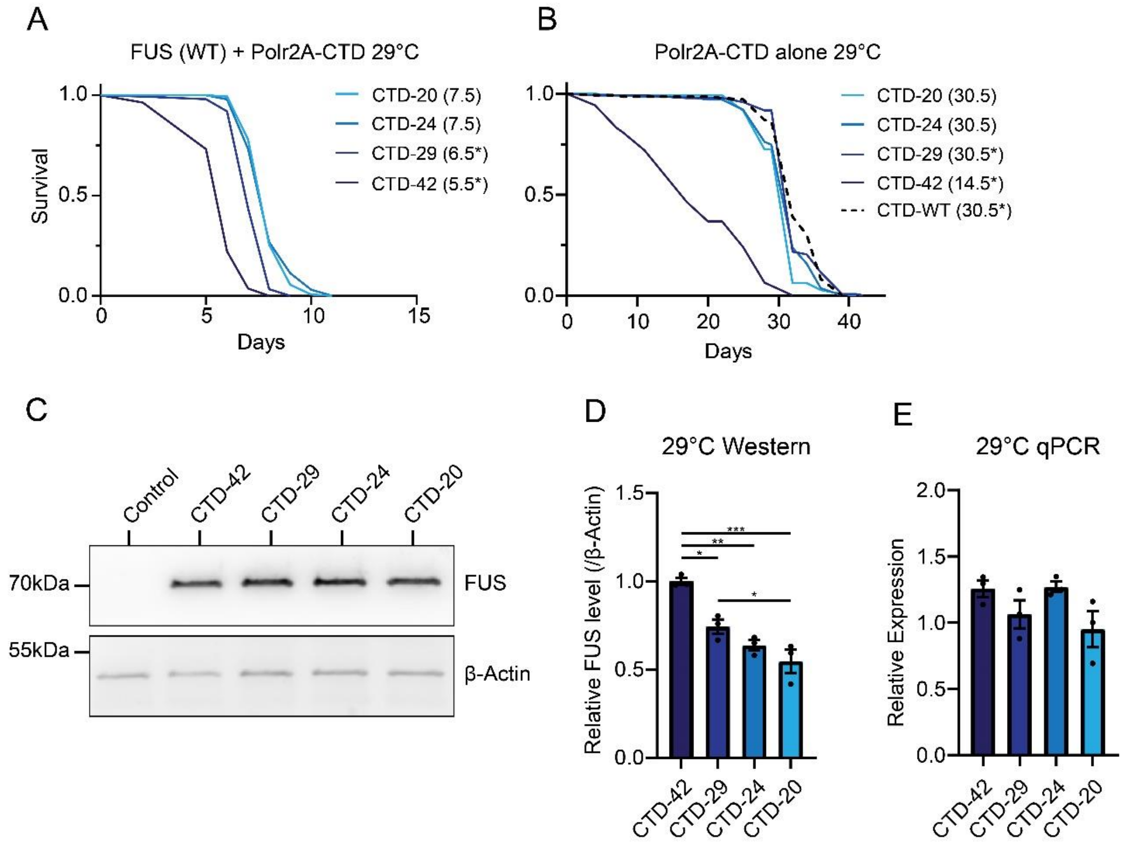

Supplementary Figure 3, related to Figure 4. A) Lifespans of males expressing FUS pan-neuronally with expression induced after development at 29°C. The length of the CTD of Polr2A is indicated. Median lifespan is shown in brackets. *Significant (P<0.05) log-rank test in comparison to CTD-20. n=97-147 per group. Full n-numbers and details of statistical tests are given in Supplementary Tables 8 and 9 **B)** Lifespan of flies carrying the modified Polr2A alone at 29°C. *Significant (P<0.05) log-rank test in comparison to CTD-20. n=125-149 per group. Full n-numbers and details of statistical tests are given in Supplementary Tables 10 and 11. **C)** Representative western blot of FUS expression in flies with different CTD lengths when lifespan is performed at 29°C. **D)** Quantification of FUS protein abundance after 6 days at 29°C. One way ANOVA (F (3, 8) = 21,25, P=0.0004). ***P<0.001, **P<0.01, **P<0.05 Tukey's multiple comparisons. n=3 per condition. Bars are mean ± SEM, individual data points are shown. **E)** Quantification of FUS transcript expression using qPCR with primers against the QGSY domain of FUS. One way ANOVA (F (3, 8) = 2,711, P=0.1153). n=3-4 per condition. Bars are mean ± SEM, individual data points are shown.

Supplementary Movie Legends:

Supplementary Movie 1, related to Figure 3A. Expression of wild-type mGFP-tagged FUS (mGFP-FUS) in salivary gland nuclei. GFP signal is pseudo-coloured using the FIRE look up table. Scale bar = 10μm. Timestamp is in seconds.

Supplementary Movie 2, related to Figure 3A. Expression of nuclear localised mGFP (mGFP-NLS) in salivary gland nuclei. GFP signal is pseudo-coloured using the FIRE look up table. Scale bar = 10μm. Timestamp is in seconds.

Supplementary Movie 3, related to Figure 3B. Expression of wild-type mGFP-tagged FUS (mGFP-FUS) in salivary gland nuclei before and after photobleaching of the region indicated in Figure 3B. GFP signal is pseudo coloured using the FIRE look up table. Scale bar = 10μm. Timestamp is in seconds.

Supplementary Table 1: Full n-numbers, and p-values for Figure 2A

Condition	Median Lifespan (Days)	n	p-value (log rank test) Control	FUS WT	FUS ΔNLS
Control	41.0	144	-	**1.6954E-60**	**2.8282E-60**
FUS WT	8.0	89	-	-	**3.0504E-58**
FUS ΔNLS	29.0	142	-	-	-

Supplementary Table 2: Raw survival data for Figure 2A.

	Control		FUS WT		FUS ΔNLS	
Day	Number Alive	Proportion Alive	Number Alive	Proportion Alive	Number Alive	Proportion Alive
0	144	1.000	89	1.000	142	1.000
2	141(3)	1.000	86(3)	1.000	138(3)	0.993
5	140	0.993	85	0.988	137	0.986
7	140	0.993	76	0.884	137	0.986
9	140	0.993	40	0.465	137	0.986
10	140	0.993	13	0.151	137	0.986
12	139	0.986	1	0.012	136(1)	0.986
14	139	0.986	0	0.000	135(1)	0.986
16	139	0.986	0	0.000	135	0.986
19	139	0.986	0	0.000	135	0.986
21	139	0.986	0	0.000	134	0.978
23	139	0.986	0	0.000	129	0.942
26	139	0.986	0	0.000	86	0.628
28	139	0.986	0	0.000	78	0.570
30	139	0.986	0	0.000	56	0.409
33	135	0.957	0	0.000	28	0.204
35	133	0.943	0	0.000	12	0.088
37	128	0.908	0	0.000	3	0.022
40	77	0.546	0	0.000	0	0.000
42	50	0.355	0	0.000	0	0.000
44	31	0.220	0	0.000	0	0.000
47	2	0.014	0	0.000	0	0.000
49	2	0.014	0	0.000	0	0.000
51	0	0.000	0	0.000	0	0.000

Numbers in brackets indicate censored flies (see methods).

Supplementary Table 3: Full n-numbers, and p-values for Figure 4B.

Condition	Median Lifespan (Days)	n	p-value (log rank test) CTD-20	CTD-24	CTD-29	CTD-42
CTD-20	29.0	121	-	**5.12E-05**	**1.31E-23**	**1.48E-53**
CTD-24	27.0	112	-	-	**5.27E-09**	**2.54E-53**
CTD-29	24.5	141	-	-	-	**1.38E-58**
CTD-42	17.5	142	-	-	-	-

Supplementary Table 4: Raw survival data for Figure 4B.

	CTD-20		CTD-24		CTD-29		CTD-42	
Day	Number Alive	Proportion Alive	Number Alive	Proportion Alive	Number Alive	Proportion Alive	Number Alive	Proportion Alive
0	121	1.000	112	1.000	141	1.000	142	1.000
2	121	1.000	112	1.000	141	1.000	141	0.993
5	120(1)	1.000	111(1)	1.000	141	1.000	139	0.979
7	119	0.992	109(2)	1.000	140	0.993	131	0.923
9	118(1)	0.992	109	1.000	140	0.993	126	0.887
12	116	0.975	108(1)	1.000	138(1)	0.986	113	0.796
14	115(1)	0.975	108	1.000	138	0.986	101	0.711
16	114	0.966	108	1.000	138	0.986	82	0.577
19	114	0.966	106(1)	0.991	136	0.972	15	0.106
21	114	0.966	106	0.991	133	0.950	6	0.042
23	114	0.966	105	0.981	126	0.900	2	0.014
26	97	0.822	57	0.533	30	0.214	0	0.000
28	82	0.695	43	0.402	18	0.129	0	0.000
30	55	0.466	24	0.224	7	0.050	0	0.000
33	22	0.186	9	0.084	0	0.000	0	0.000
35	11	0.093	5	0.047	0	0.000	0	0.000
37	8	0.068	4	0.037	0	0.000	0	0.000
40	2	0.017	0	0.000	0	0.000	0	0.000
42	0	0.000	0	0.000	0	0.000	0	0.000

Numbers in brackets indicate censored flies (see methods).

Supplementary Table 5: Full n-numbers, and p-values for Figure 4C.

			p-value (log rank test)				
Condition	Median Lifespan (Days)	n	CTD-20	CTD-24	CTD-29	CTD-42	CTD-WT
CTD-20	49.0	135	-	**2.03E-04**	**7.01E-09**	**8.18E-32**	**8.35E-24**
CTD-24	51.5	136	-	-	1.40E-01	**2.49E-40**	**3.72E-13**
CTD-29	55.5	140	-	-	-	**4.15E-48**	**7.62E-11**
CTD-42	26.5	142	-	-	-	-	**2.55E-55**
CTD-WT	65.5	136	-	-	-	-	-

Supplementary Table 6: Raw survival data for Figure 4C

	CTD-WT		CTD-20		CTD-24		CTD-29		CTD-42	
Day	Number Alive	Proportion Alive	Number Alive	Proportion Alive	Number Alive	Proportion Alive	Number Alive	Proportion Alive	Number Alive	Proportion Alive
0	136	1.000	135	1.000	136	1.000	140	1.000	142	1.000
4	135	0.993	134	0.993	135(1)	1.000	139	0.993	140	0.986
7	135	0.993	134	0.993	134	0.993	138(1)	0.993	137	0.965
8	135	0.993	134	0.993	134	0.993	137(1)	0.993	134	0.944
11	134(1)	0.993	134	0.993	132(1)	0.985	137	0.993	121	0.852
14	134	0.993	134	0.993	130(1)	0.978	137	0.993	113	0.796
18	134	0.993	133	0.985	129	0.970	137	0.993	104	0.732
20	134	0.993	132	0.978	129	0.970	137	0.993	96	0.676
22	133	0.985	132	0.978	128(1)	0.970	137	0.993	92	0.648
25	132	0.978	132	0.978	127	0.963	137	0.993	77	0.542
28	131	0.970	130(1)	0.970	127	0.963	136	0.986	68	0.479
29	130	0.963	130	0.970	127	0.963	136	0.986	65	0.458
33	128(1)	0.956	126	0.941	125	0.947	135	0.978	33	0.232
36	127	0.948	121	0.903	123	0.932	132	0.957	23	0.162
39	127	0.948	116	0.866	120	0.910	129	0.935	19	0.134
41	126	0.941	109	0.814	113(1)	0.864	126	0.913	17	0.120
43	126	0.941	102	0.761	111	0.849	124	0.899	16	0.113
46	125	0.933	80	0.597	104	0.795	119	0.862	9	0.063
48	115	0.859	68	0.508	87	0.665	113(1)	0.826	7	0.049
50	112	0.836	64	0.478	83	0.635	106	0.775	6	0.042
53	103	0.769	41	0.306	65	0.497	90	0.658	6	0.042
54	102	0.762	32	0.239	62	0.474	85	0.621	6	0.042
57	94(1)	0.709	26	0.194	53(1)	0.413	65	0.475	6	0.042
60	83	0.626	10	0.075	26	0.203	35	0.256	0	0.000
62	79	0.596	9	0.067	22	0.171	32	0.234	0	0.000
64	73	0.551	9	0.067	18	0.140	26	0.190	0	0.000
67	55	0.415	4	0.030	11	0.086	16	0.117	0	0.000
69	50	0.377	2	0.015	7	0.055	10	0.073	0	0.000
71	43	0.324	1	0.007	6	0.047	7	0.051	0	0.000
74	27	0.204	1	0.007	4	0.031	3	0.022	0	0.000
76	23	0.174	1	0.007	4	0.031	1	0.007	0	0.000
78	19	0.143	1	0.007	3	0.023	1	0.007	0	0.000
81	7	0.053	1	0.007	0	0.000	1	0.007	0	0.000
83	3	0.023	1	0.007	0	0.000	1	0.007	0	0.000
85	3	0.023	1	0.007	0	0.000	1	0.007	0	0.000
88	3	0.023	0	0.000	0	0.000	1	0.007	0	0.000
90	2	0.015	0	0.000	0	0.000	1	0.007	0	0.000
92	2	0.015	0	0.000	0	0.000	0	0.000	0	0.000
95	0	0.000	0	0.000	0	0.000	0	0.000	0	0.000

Numbers in brackets indicate censored flies (see methods).

Supplementary Table 7: Information on post-mortem material.

Disease status	Sex	Age (years)	PM Delay (hours)	POLR2A Inclusions
FTLD-FET	M	40	32	Yes
FTLD-FET	M	53	72	Yes
FTLD-FET	M	60	48	No
ALS-FUS (R495*)	F	34	38	No
ALS-FUS (K510E)	M	39	51	No
Control	M	97	25	No
Control	M	74	22.5	No
Control	F	99	45.5	No

Supplementary Table 8: Full n-numbers, and p-values for Supplementary Figure 3A

			p-value (log rank test)			
Condition	Median Lifespan (Days)	n	CTD-20	CTD-24	CTD-29	CTD-42
CTD-20	7.5	146	-	6.02E-01	**2.45E-13**	**1.72E-46**
CTD-24	7.5	97	-	-	**4.61E-10**	**1.52E-33**
CTD-29	6.5	149	-	-	-	**1.66E-30**
CTD-42	5.5	108	-	-	-	-

Supplementary Table 9: Raw survival data for Figure 3A

	CTD-20		CTD-24		CTD-29		CTD-42	
Days	Number Alive	Proportion Alive	Number Alive	Proportion Alive	Number Alive	Proportion Alive	Number Alive	Proportion Alive
0	146	1.000	97	1.000	149	1.000	108	1.000
2	145(1)	1.000	97	1.000	148	0.993	104	0.963
5	145	1.000	97	1.000	146	0.980	79	0.731
6	144	0.993	95	0.979	137	0.919	24	0.222
7	113	0.779	71	0.732	65	0.436	4	0.037
8	37	0.255	26	0.268	5	0.034	0	0.000
9	8	0.055	11	0.113	0	0.000	0	0.000
10	1	0.007	3	0.031	0	0.000	0	0.000
11	0	0.000	0	0.000	0	0.000	0	0.000

Numbers in brackets indicate censored flies (see methods).

Supplementary Table 10: Full n-numbers, and p-values for Supplementary Figure 3B

			p-value (log rank test)				
Condition	Median Lifespan (Days)	n	CTD-20	CTD-24	CTD-29	CTD-42	CTD-WT
CTD-20	30.5	146	-	7.06E-02	**6.84E-07**	**4.02E-43**	**1.27E-07**
CTD-24	30.5	144	-	-	**1.71E-03**	**6.38E-43**	**7.49E-04**
CTD-29	30.5	148	-	-	-	**4.375E-55**	8.53E-01
CTD-42	14.0	125	-	-	-	-	**3.04E-53**
CTD-WT	30.5	149	-	-	-	-	-

Supplementary Table 11: Raw survival data for Figure 3B

	CTD-WT		CTD-20		CTD-24		CTD-29		CTD-42	
Days	Number Alive	Proportion Alive	Number Alive	Proportion Alive	Number Alive	Proportion Alive	Number Alive	Proportion Alive	Number Alive	Proportion Alive
0	149	1.000	146	1.000	144	1.000	148	1.000	125	1.000
4	148	0.993	146	1.000	141(2)	0.993	147	0.993	118	0.944
7	147(1)	0.993	144(1)	0.993	140(1)	0.993	147	0.993	104	0.832
8	146	0.987	144	0.993	139(1)	0.993	147	0.993	101	0.808
11	146	0.987	144	0.993	137(1)	0.986	147	0.993	90	0.720
17	145(1)	0.987	144	0.993	137	0.986	144(1)	0.980	58	0.464
20	145	0.987	144	0.993	135	0.972	144	0.980	46	0.368
22	144	0.980	144	0.993	135	0.972	144	0.980	46	0.368
25	142(1)	0.973	133	0.917	128	0.921	141	0.959	30	0.240
28	127	0.870	105	0.724	106	0.763	135	0.918	8	0.064
29	126	0.863	105	0.724	104	0.748	135	0.918	6	0.048
32	57	0.391	9	0.062	33	0.237	32	0.218	0	0.000
34	44	0.301	9	0.062	22	0.158	30	0.204	0	0.000
36	12	0.082	4	0.028	5	0.036	17	0.116	0	0.000
39	0	0.000	1	0.007	0	0.000	1	0.007	0	0.000
41	0	0.000	0	0.000	0	0.000	1	0.007	0	0.000
42	0	0.000	0	0.000	0	0.000	0	0.000	0	0.000

Additional References:

1 Buhler K, Clements J, Winant M, Bolckmans L, Vulsteke V, Callaerts P. Growth control through regulation of insulin signalling by nutrition-activated steroid hormone in Drosophila. *Development* 2018; **145**: dev165654.
